# Decoding Buried Interfaces in Perovskite Solar Cells: Core Issues, Strategic Engineering, and Prospects for High‐Efficiency Stable Devices

**DOI:** 10.1002/advs.202512523

**Published:** 2025-09-14

**Authors:** Peng Mao, Weihui Bi, Jun Lv, Zongbao Zhang, Bing Wang, Yufei Zhong

**Affiliations:** ^1^ Zhejiang engineering research center for fabrication and application of advanced photovoltaic materials School of Materials Science and Engineering NingboTech University No.1 Qianhu South Road Ningbo 315100 P. R. China; ^2^ Zhejiang Engineering Research Center for Fabrication and Application of Advanced Photovoltaic Materials Institute for Carbon Neutrality Ningbo Innovation Centre Zhejiang University Ningbo 315048 P. R. China; ^3^ Nanjing University Nanjing Jiangsu 210008 P. R. China; ^4^ Chair for Emerging Electronic Technologies Technical University of Dresden Nöthnitzer Str. 61 01187 Dresden Germany; ^5^ Leibniz‐Institute for Solid State and Materials Research Dresden Helmholtzstraße 20 01069 Dresden Germany

**Keywords:** buried interfaces, issues, interfacial modification, perovskite solar cells, stability

## Abstract

Perovskite solar cells (PSCs) have emerged as a frontrunner in photovoltaic technologies, owing to their high performance and low‐cost scalability. However, their efficiency remains substantially below the theoretical Shockley‐Queisser limit (>30%), and their long‐term stability is severely compromised; both are predominantly driven by interface‐related issues. Compared to the top interface, buried interfaces are equally important, if not more important, effects on perovskite film quality and device performance. This review comprehensively analyzes challenges at buried interfaces, including defects, terminations, strain, carrier dynamics, and chemical reactions, with special focus on self‐assembled monolayer (SAM)‐based devices and textured interfaces in perovskite/silicon tandem solar cells. Targeted modification strategies such as defect passivation, strain control, carrier transport modulation, and inhibition of adverse reactions are proposed to mitigate these issues. Finally, research prospects for optimizing buried interfaces are outlined, including advanced in situ characterizations, novel charge transport materials, and innovative interface engineering to enhance PSC performance and stability.

## Graphical abstract

This review offers a thorough overview of the diverse buried interfaces in PSCs, emphasizing the associated issues, such as defects, perovskite terminations, strain, carrier dynamics, and chemical reactions. Moreover, the buried interface issues in both the widely studied SAM‐based devices and perovskite/silicon tandem solar cells are discussed in detail. Lastly, it suggests a range of modification strategies to tackle these challenges effectively.

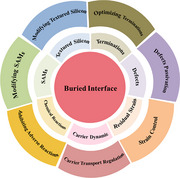

## 1 Introduction

In recent years, the realm of semiconducting optoelectronic materials has witnessed the rise of halide perovskites, which have rapidly garnered substantial attention and catalyzed extensive research endeavors.^[^ ^1^, ^2^, ^3^, ^4^ ^]^ Endowed with exceptional photoelectronic properties, perovskites find widespread applications in optoelectronic devices, spanning photovoltaics, light‐emitting diodes (LEDs), lasers, and X‐ray detectors.^[^ ^5^, ^6^, ^7^, ^8^, ^9^, ^10^ ^]^ Of particular note are perovskite solar cells (PSCs), the most intensively studied perovskite‐based devices, boasting a remarkable power conversion efficiency (PCE) exceeding 27% to date.^[^ ^1^, ^11^, ^12^, ^13^, ^14^, ^15^ ^]^ Nevertheless, there remains ample room for improvement when compared to their Shockley‐Queisser (S‐Q) theoretical efficiency limit (>30%). Furthermore, these devices suffer from severe long‐term stability issues, primarily stemming from trap‐assisted non‐radiative recombination losses and photochemical degradation—phenomena that are especially pronounced at the interfaces between perovskites and charge transport layers (CTLs).^[^ ^16^ ^]^ As such, the precise regulation of the interface between perovskite films and CTLs, namely interface engineering/modification, is of paramount importance. This direct correlation between interface quality and device performance has been validated by numerous studies,^[^ ^17^, ^18^ ^]^ highlighting the critical need for delicate regulation of these interfaces.

In general, PSCs feature two critical interfacial contacts: the perovskite top surface and the buried interface. Given the relative ease of controlling the exposed top surface compared to the buried bottom surface, post‐treatment of the exposed top surface has attracted considerable attention in recent years.^[^ ^19^, ^20^, ^21^ ^]^ Compared to the top surface, the buried interface has distinct characteristics. It directly dictates perovskite nucleation and growth, governing crystalline quality and defect distribution, which are the fundamental properties that the top surface (formed later) cannot alter. As the primary site for initial charge separation, it more profoundly impacts carrier dynamics and long‐term stability, whereas the top surface mainly affects surface recombination and environmental interactions (e.g., moisture). In this regard, buried interface studies will not only enhance the performance of existing devices but also will lay the groundwork for the development of more efficient and stable PSC technologies moving forward. Therefore, this by no means implies that the buried interface can be overlooked; optimizing the perovskite buried interface is equally crucial to the top surface.

Notably, the buried interface in PSCs constitutes a highly complex and dynamic system involving a variety of interdependent factors, including terminations, interfacial defects,^[^ ^22^, ^23^ ^]^ carrier transfer,^[^ ^24^, ^25^ ^]^ residual strain,^[^ ^26^ ^]^ and interfacial chemical reactions.^[^ ^27^, ^28^ ^]^ Interfacial defects, which typically arise during fabrication due to non‐ideal conditions such as poor crystallinity, inferior grain growth, and inadequate interface quality, create localized states within the bandgap. These defects can trap carriers, impede their movement, and induce recombination, thereby compromising device performance.^[^ ^29^, ^30^ ^]^ In PSCs, efficient carrier transfer across interfaces is essential for generating photocurrent and producing electricity. Obstacles to carrier transfer, such as energy level mismatches, can lead to energy losses and ultimately reduce device efficiency.^[^ ^31^, ^32^ ^]^ Moreover, the matching of lattice constants between the buried CTLs and the perovskite plays a role in determining the quality of perovskite films. Lattice mismatches can result in residual strain, which profoundly affects the optoelectronic properties of the perovskite.^[^ ^33^ ^]^ Apart from the aforementioned factors, the quality of the perovskite is also influenced by the chemical properties of the CTLs. Chemically active CTLs can trigger degradation reactions in perovskite, thereby undermining the chemical stability of PSCs.^[^ ^34^ ^]^ It is noteworthy that self‐assembled monolayers (SAMs), widely used buried hole selective materials, suffer from issues including poor wettability, facile molecular clustering, and incomplete substrate coverage. Additionally, in perovskite/silicon tandem solar cells (P/S‐TSCs), the textured silicon interface embedded in the substrate can affect the deposition of the perovskite layer, leading to non‐conformal growth. Given this complexity, a comprehensive examination of the buried interface is imperative to address these intertwined issues and realize high‐efficiency, stable PSCs.

This review aims to provide a thorough overview of the buried interface in PSCs. It examines several issues associated with the buried interface, including interface defects, perovskite terminations, residual strain, carrier dynamics, and interface degradation reactions. Furthermore, the buried interface challenges in SAM‐based devices and the textured interface obstacles in P/S‐TSCs also present critical engineering hurdles. To address these issues, commonly developed interfacial optimization methods are summarized. These methods seek to improve interface quality, minimize defects, reduce residual strain, and enhance carrier transport across the buried interface—ultimately leading to improvements in the performance and stability of PSCs. Finally, future advances in PSCs are outlined, including novel CTLs, advanced in situ characterization, and innovative interface engineering.

## 2 Diversity of Buried Interface

### 2.1 Composition of Perovskites

The buried interface in PSCs is a complex system, comprising a variety of perovskite components and the use of CTLs. These different types of perovskite components and CTLs can interact in complex ways, leading to a diverse range of buried interface properties and behaviors. Gaining an understanding of this interface through applying a unified theory or mechanism is a significant challenge. Regardless of their compositions (organic–inorganic hybrid or all‐inorganic systems), perovskite active layers can exhibit significant surface termination features of organic molecular fractions or metal halide moieties.^[^ ^17^, ^35^ ^]^ These features directly affect the surface potential, electronic structure, and chemical reactivity. This effect becomes even more pronounced when simple molecular components such as methylammonium (MA) or formamidinium (FA) are replaced by long‐chain cations like butylammonium (BA)^[^ ^36^ ^]^ or phenethylammonium (PEA).^[^ ^37^, ^38^ ^]^ The state‐of‐the‐art perovskite devices are based on FAPbI_3_ to date, which includes partial substitution of cesium (Cs) and MA at the A‐position and bromine at the X‐position,^[^ ^39^, ^40^, ^41^ ^]^ and are currently the subject of extensive investigation. This complex perovskite component can result in numerous buried interfaces within the PSCs. Notably, the selection of buried interfaces for various A‐site‐based perovskites differs based on their distinct chemical stabilities, crystallization behaviors, and lattice characteristics, guiding principles that align with their unique material properties to optimize interface compatibility. Understanding these interactions is crucial for designing and optimizing PSCs to achieve better performance and stability.

### 2.2 Charge Transport Layers

#### 2.2.1 Electron Transport Layers in Regular PSCs

**Figure 1:**
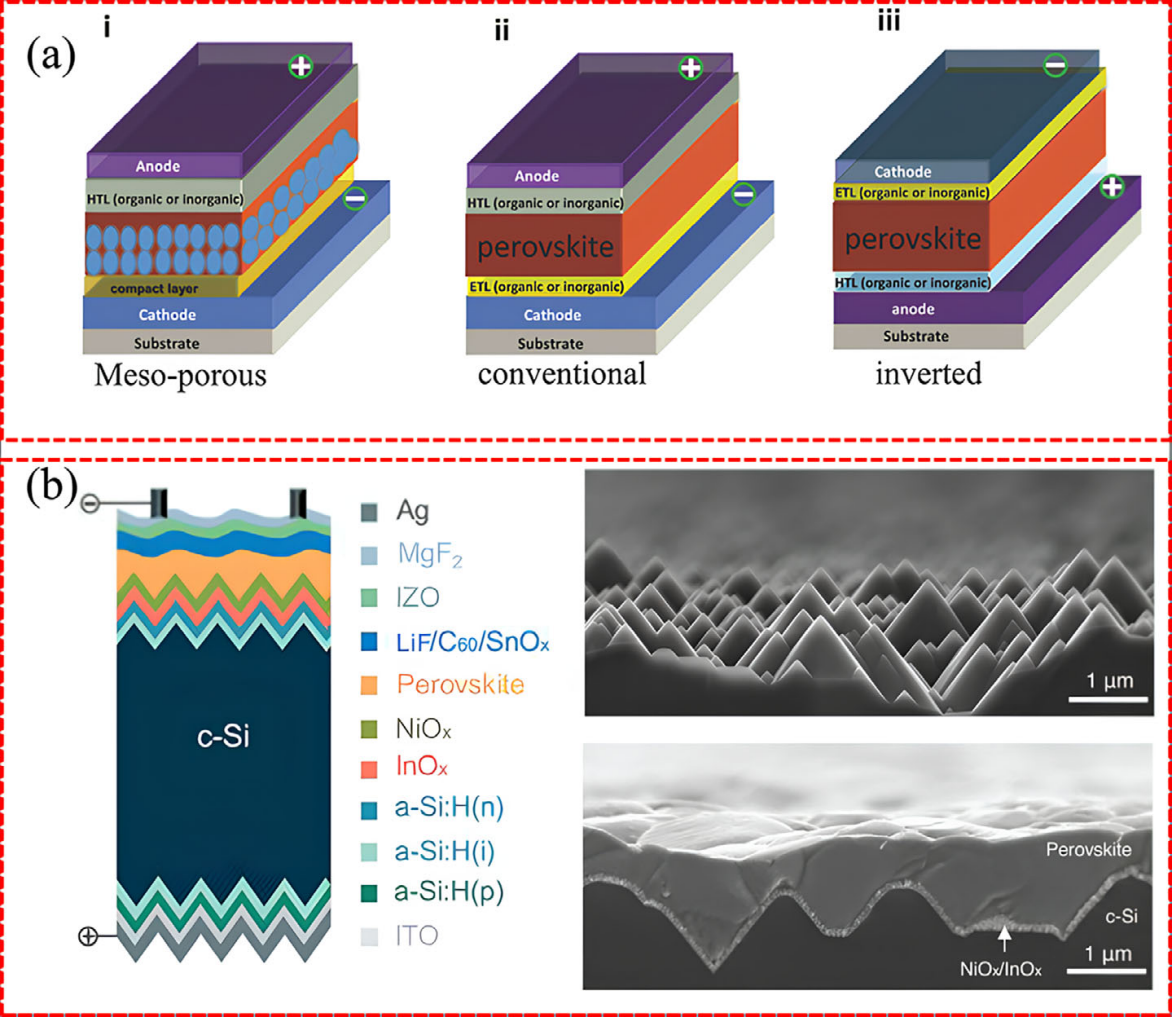
a) Basic structures of PSCs: (i) mesoporous structure with cathode/compact (TiO_2_)/mesoporous layer (TiO_2_ or Al_2_O_3_)/perovskite/HTL/anode, (ii) conventional structure with cathode/ETL/perovskite/HTL/anode, and (iii) inverted structure with anode/HTL/perovskite/ETL/cathode. Reproduced with permission.^[^ ^44^ ^]^ Copyright 2018, Wiley‐VCH. b) Device structure of a 2T P/S TSC. The perovskite layer is deposited by solution‐processed on a double‐side textured Si bottom cell. The cross‐section SEM images show the textured Si with pyramid morphology, and it is fully covered by a perovskite top cell with a thick perovskite film. Reproduced with permission.^[^ ^75^ ^]^ Copyright 2020, American Association for the Advancement of Science.

In addition to perovskite species, the diversity of buried interfaces is influenced by the characteristics of adjacent CTLs. As illustrated in **Figure** **1**a, the buried CTLs in PSCs can be broadly classified into two types depending on the carrier type, namely electron transport layers (ETLs) for the regular (n–i–p) structures and hole transport layers (HTLs) for the inverted (p–i–n) structure.^[^ ^42^, ^43^, ^44^ ^]^ In a regular PSC, titanium dioxide (TiO_2_), zinc oxide (ZnO), and tin oxide (SnO_2_) are the most frequently used electron transport materials because of their high electron mobility and suitable energy level alignment with perovskite.^[^ ^45^, ^46^, ^47^ ^]^ TiO_2_ was first used as an electron transport material in PSCs and can be employed in various forms, including nanoparticles,^[^ ^48^, ^49^ ^]^ mesoporous films,^[^ ^50^, ^51^ ^]^ and compact layers,^[^ ^52^, ^53^, ^54^ ^]^ making it a versatile material that can be tailored to different device architectures and processing techniques. However, its further development is hampered by the need for high‐temperature processing and its sensitivity to ultraviolet light.^[^ ^55^, ^56^ ^]^ Another candidate is ZnO, which has a wide bandgap and is suitable for scalable manufacturing. However, it contains defects or impurities (e.g., hydroxyl, OH^−^) that act as trap states for charge carriers and nucleation sites for the formation of lead iodide (PbI_2_) or other perovskite decomposition products.^[^ ^28^, ^57^ ^]^ Moreover, Zn^2+^ can diffuse into the perovskite layer and cause degradation.^[^ ^58^ ^]^ Recently, SnO_2_ has been widely used due to its facile solution preparation and suitable compatibility with perovskite.^[^ ^59^, ^60^ ^]^ SnO_2_ can form better contact with the perovskite layer than TiO_2_ and ZnO, enabling efficient charge extraction and reducing the possibility of defects or recombination.

#### 2.2.2 Hole Transport Layers in Inverted PSCs

In inverted PSCs, the hole transport materials are deposited directly onto transparent conductive oxide glass to extract holes from the perovskite film. Common HTL materials comprise poly(3,4‐ethylenedioxythiophene):polystyrene sulfonate (PEDOT:PSS),^[^ ^61^ ^]^ poly[bis(4‐phenyl)(2,4,6‐trimethylphenyl)amine] (PTAA),^[^ ^62^, ^63^ ^]^ nickel oxide (NiO_x_),^[^ ^64^, ^65^ ^]^ and SAMs‐based on carbazole and phosphonic acid groups (nPACz, where n is the aliphatic chain length) and their derivatives.^[^ ^66^, ^67^ ^]^ PEDOT:PSS exhibits high conductivity and is compatible with most perovskite materials, forming a good interface with the perovskite layer.^[^ ^68^ ^]^ However, its acidity and low work function limit the enhancement of device performance.^[^ ^69^, ^70^, ^71^ ^]^ PTAA, with its high hole mobility and good stability, has the potential to achieve high efficiency, but the complicated synthesis and poor wettability pose challenges.^[^ ^72^ ^]^ NiO_x_ is a low‐cost p‐type metal oxide with tunable optoelectronic properties, facilitating the formation of better ohmic contact with the perovskite layer due to its deep valence band.^[^ ^73^ ^]^ Nevertheless, numerous impurities and surface defects often appear in the NiO_x_ films, leading to hole accumulation at the perovskite interface, high charge carrier recombination, and acceleration of PSC degradation.^[^ ^27^ ^]^ Recently, PSCs have achieved remarkable efficiencies exceeding 26% with excellent stability by using SAMs of carbazole‐based molecules with phosphonic acid anchoring groups (such as 2PACz, MeO‐2PACz, and Me‐4PACz) as HTLs.^[^ ^66^, ^74^ ^]^ This success can be attributed to three main factors: reduced resistance through ultrathin SAMs, efficient hole extraction facilitated by an ordered molecular arrangement, and minimized charge carrier recombination at the buried interface.

### 2.3 Buried Interface of P/S TSCs

To enhance the utilization of long‐wavelength photons, PSCs can be paired with bottom cells featuring intermediate or narrow bandgap, such as P/S‐TSCs technology. As illustrated in Figure 1b, in the common two‐terminal (2T) structure, the top PSC is directly grown on the bottom silicon solar cell.^[^ ^75^ ^]^ At present, the photoelectric conversion efficiency of single‐junction P/S TSCs has exceeded 34%,^[^ ^15^ ^]^ highlighting their potential as a future high‐performance photovoltaic technology. Over recent years, efforts have focused on depositing perovskite on textured silicon cells, as this approach can significantly reduce reflection of incident photons and extend the propagation path of photons within the absorption layer.^[^ ^76^, ^77^, ^78^ ^]^ Numerical studies have underscored the significance of introducing textured device interfaces to achieve effective light management. Uniform deposition of perovskite materials onto industrially viable textured silicon solar cells can not only reduce manufacturing costs but also enhance the matched photoelectric current density. For silicon solar cells, random cone‐shaped textures with sizes of a few micrometers are commonly employed for light management. However, perovskite films grown on fully textured silicon often exhibit poor crystalline quality, which can degrade photovoltaic performance. Notably, such textured silicon surfaces cannot deliver optimal performance unless further tailored to be compatible with solution‐processed perovskite absorption layers.^[^ ^79^ ^]^

## 3 Issues Associated with the Buried Interface

### 3.1 Perovskite Terminations

The perovskite terminations and their influence on the buried interface present significant issues. Despite the significant progress in techniques for depositing perovskite films, achieving uniform perovskite films remains a persistent challenge. The surfaces of perovskite films frequently deviate from ideal stoichiometry and exhibit defects, creating favorable circumstances for the presence of surface terminations. Before delving into the specific terminations found on different perovskite surfaces, it is important to establish a theoretical foundation by describing the stoichiometric surface of perovskites using density functional theory (DFT) slab calculations. This approach aims to provide a thorough understanding of the surface, including its unique properties and its influence on adjacent functional layers.

Early studies focused on investigating the structural stability and electronic states of the representative (110), (001), (100), and (101) surfaces of the tetragonal MAPbI_3_ using DFT.^[^ ^80^ ^]^ The calculation reveals that the vacant terminations (i.e., MAI‐rich) present higher stability than flat terminations (i.e., PbI_2_‐rich) on the surfaces by comparing various types of PbI_x_ polyhedra under thermodynamic equilibrium conditions. Both MAI‐rich and PbI_2_‐rich surface terminations can coexist on the energetically favorable (110) and (001) surfaces of tetragonal MAPbI_3_ due to their similar formation enthalpies. Furthermore, it was found that the PbI_2_‐rich terminations on the (110) and (001) surfaces exhibit shallow surface states that effectively facilitate hole transfer. It should be mentioned that the dominance of either MAI‐rich termination or PbI_2_‐rich termination on the tetragonal MAPbI_3_ surface depends on the specific process conditions during the film deposition.

Furthermore, studies have shown that the termination of perovskite affects the energy level alignment of the perovskite layer. Quarti et al. investigated the energies of the frontier crystal orbitals of the MAI‐terminated and PbI_2_‐terminated surfaces through DFT calculations on slab models of the (001) surface.^[^ ^81^ ^]^ They found that in the case of MAPbI_3_ perovskite, surface termination affects electron density distribution, leading to surface dipoles with opposite directions and subsequent shifts in the vacuum level. Specifically, PbI‐terminated surfaces expose electron‐enriched layers, while MAI‐terminated surfaces present electron‐tapered layers, as indicated by planar‐averaged electron density calculations. Bader charge analysis confirms electron transfer from MAI to PbI layers, resulting in surface dipoles pointing in opposite directions for the two terminations. According to the Helmholtz equation:

(1) $$\Delta \mathrm{vac}=-\frac{\mu }{\varepsilon_{0}A}$$

(here µ denotes the surface dipole component perpendicular to the surface, ε_0_ stands for the vacuum dielectric constant, A refers to the surface area, and Δvac represents the displacement of the vacuum level), these dipoles cause distinct vacuum level shifts: the MAI‐terminated surface induces an upshift of the vacuum level, whereas the PbI‐terminated surface leads to a downshift. Therefore, the PbI_2_‐terminated surface exhibited VBM and CBM ≈1 eV lower than the MAI‐terminated surface. This difference in energy levels has a substantial effect on the energy alignment with the adjacent CTLs in PSCs.

For the widely studied FAPbI_3_, S.M. Oner et al. calculated the surface energies of four possible termination types^[^ ^82^ ^]^: PbI_2_, FAI, I, and PbI. When compared to PbI and I terminations, the FAI and PbI_2_ terminations exhibit lower surface energies and higher defect formation energies. The lower surface energy is indicative of more stable structural configurations, which are inherently more resistant to defect formation. In addition, FAI termination specifically exhibits the lowest surface energy among the four, further underscoring its highest stability in this context. Therefore, FAI termination is optimal for stability and photovoltaic performance due to its low surface energy, high defect formation energy, and shallow charge transfer levels. However, high volatility and environmental sensitivity (to heat/humidity) of FA molecules may cause abandonment of FAI termination, potentially transforming into I termination and leading to unstable surfaces prone to defects. PbI_2_ termination is another candidate, as its surface energy ranks second lowest. Recent studies show controlled PbI_2_ termination can improve device performance due to a suitable bandgap and the absence of volatile FA.^[^ ^17^ ^]^ On PbI_2_ surfaces, electron‐donating FA_i_ and PbI defects dominate in hole‐rich regions (low Fermi level), suggesting that passivation treatments creating deep transition levels on PbI_2_ terminations may further enhance FAPbI_3_ solar cell performance.

**Figure 2:**
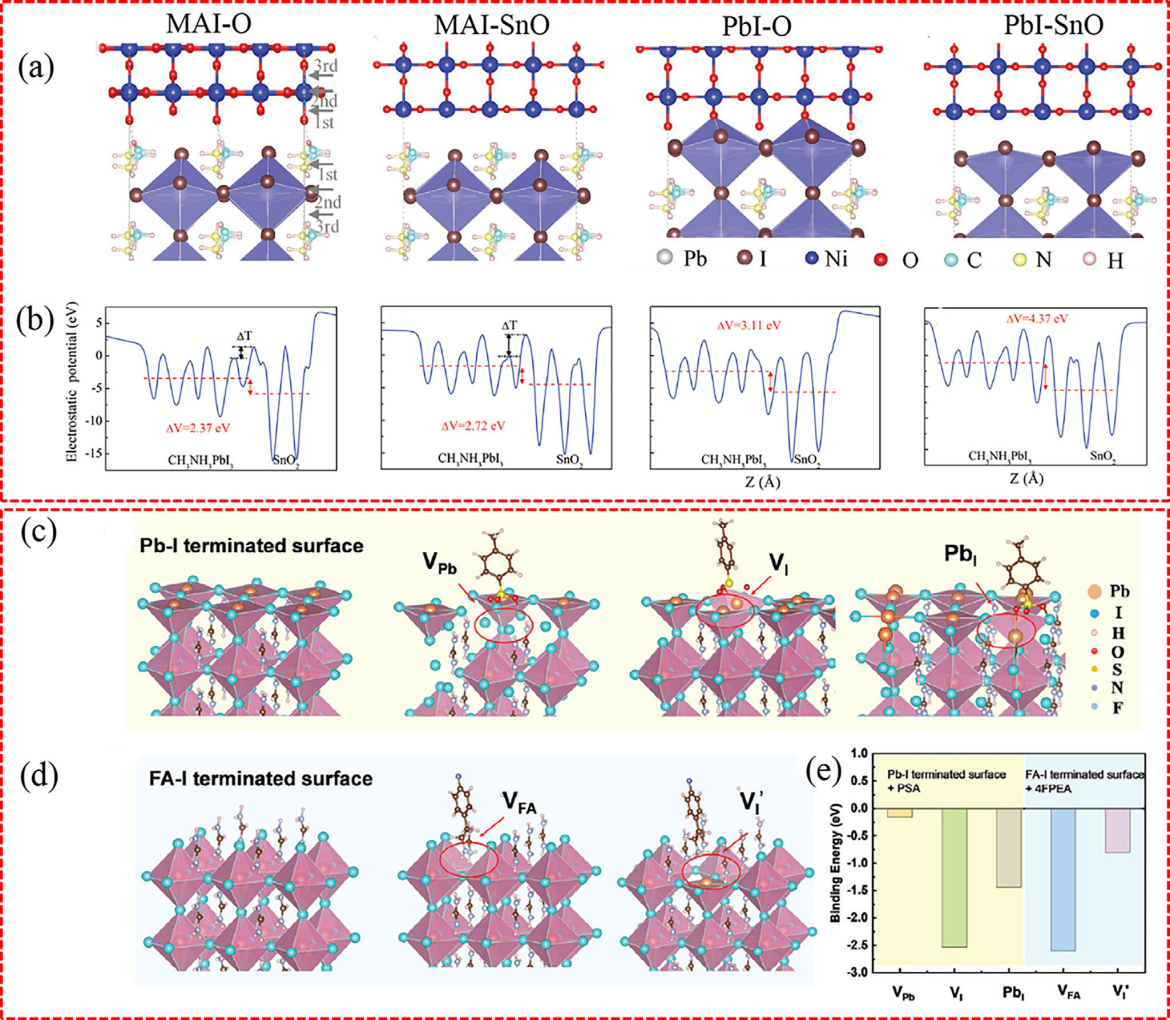
a) Geometric structures of CH_3_NH_3_PbI_3_/SnO_2_ interfaces with four configurations: MAI‐O, MAI‐SnO, PbI–O, and PbI‐SnO. b) Planar‐averaged electrostatic potential of different CH_3_NH_3_PbI_3_/SnO_2_ interfaces along the vertical *z*‐direction normal to the MAI‐O, MAI‐SnO, PbI–O, and PbI‐SnO interfaces. The electrostatic potential difference (ΔV) between CH_3_NH_3_PbI_3_ surface and the SnO_2_ surface is marked by the red arrow. a,b) Reproduced with permission.^[^ ^83^ ^]^ Copyright 2019, Wiley‐VCH. c) Schematic diagram of the Pb–I terminated surfaces without defect sites and binding interaction between PSA^−^ and Pb‐I terminated surface with the defects of V_Pb_, V_I_, and Pb_I_. d) Schematic diagram of FA‐I terminated surface without defect sites, and binding interaction between 4FPEA^+^ and FA‐I terminated surface with defects of V_FA_ and V_I_’. e) Binding energy comparison between 4FPEAPSA and various defects, including V_Pb_, V_FA_, V_I_, V_I_’, and Pb_I_. And V_I_ and V_I_’ refer to the iodine vacancy at the Pb–I and FA‐I terminated surface, respectively. c–e) Reproduced with permission.^[^ ^84^ ^]^ Copyright 2024, Wiley‐VCH.

For the buried interface, the absence of direct experimental evidence to characterize the interfacial terminations hinders the in‐depth understanding of the interaction between perovskites and CTLs. Even so, theoretical calculation may provide a compromise solution when we gain insight into the microscopic mode of the interaction. Taking SnO_2_/MAPbI_3_ as an example (**Figure** **2**a), DFT results show that both the terminations of SnO_2_ and MAPbI_3_ have significant effects on the interfacial transport properties and, consequently, the device performance.^[^ ^83^ ^]^ Specifically, the presence of the PbI‐SnO interface is proven to be more advantageous for hole blocking and electron transport. This is attributed to the largest valence band offset observed in comparison with other interfaces, such as MAI‐SnO, MAI‐O, and PbI‐O interfaces. In addition, as illustrated in Figure 2b, the electrostatic potential differences for the MAI‐O, MAI‐SnO, PbI‐O, and PbI‐SnO interfaces are 2.37, 2.72, 3.11, and 4.37 eV, respectively. Since electrons tend to transfer from a surface with a higher electrostatic potential to one with a lower potential, interfaces with PbI terminations exhibit stronger charge transfer compared to those with MAI terminations. Therefore, the PbI‐SnO interface demonstrates a more pronounced electrostatic potential difference, which directly contributes to the enhanced electron transfer efficiency. The PSC with PbI_2_ as the buried termination layer outperforms the PSC with MAI as the buried termination layer, as evidenced by an increase in PCE from 17.6% to 18.7%. In another study, in order to better understand the effect of 2‐(4‐fluorophenyl)ethylammonium‐4‐methyl benzenesulfonate (4FPEAPSA) at the buried interface on perovskite, Zhang et al. used DFT to perform calculations on PbI_2_ and FAI‐terminated surfaces.^[^ ^84^ ^]^ As shown in Figure 2c,d, they simulated the interaction between 4FPEAPSA and perovskite by pairing the PSA anions with the PbI_2_‐terminated surface and the 4FPEA cations with the FAI‐terminated surface. They observed that the S and O atoms in PSA showed a strong affinity toward Pb^2+^, resulting in the distortion of the crystal structure. At the same time, the 4FPEA^+^ cations tended to integrate into the^[^PbX_6_]^4−^ octahedral lattice within the FA vacancy defect model, which may help improve the crystallization process of the perovskite film. Figure 2e demonstrates that the binding energy analysis also showed consistent results.

The above research results indicate that, as a critical design parameter for optimizing PSCs, perovskite terminations govern energy level alignment, defect propensity, and chemical compatibility with CTLs. These terminations dynamically respond to environmental conditions and charge transport properties, affecting carrier transport efficiency and device stability. Modifiers can be employed to tune terminations, selectively mitigating defects by targeting specific termination‐related traps. However, challenges remain in the precise control of perovskite terminations.

### 3.2 Defects at the Buried Interface

Regarding improving device performance, the defects at the buried interface pose a major challenge that needs to be overcome. The buried interface defects generally originate from the surfaces of both the CTLs and the bottom surface of the perovskite layer. Due to the unique ionic properties and low defect formation energy of perovskites, high‐concentration defects (≈10^15^cm^−^^3^) can easily form when the perovskite precursor solutions rapidly transform into polycrystalline films, particularly at the film's surfaces and grain boundaries.^[^ ^85^ ^]^ The defect density at the interface is one to two orders of magnitude higher than that in the interior.^[^ ^86^ ^]^ Defects can be classified into shallow‐ and deep‐level traps based on their position in the band gap. Shallow‐level traps are located near the top of the valence band or the bottom of the conduction band and can be re‐emitted back into the conduction band minimum (CBM) or valence band maximum (VBM) by absorbing phonons. In contrast, deep‐level traps are located in the middle of the perovskite's bandgap and have a significant impact on non‐radiative recombination.^[^ ^87^ ^]^ From the following equation,

(2) $$V_{oc}=\frac{n_{ID}k_{B}T}{q}\;ln\left(\frac{J_{sc}}{J_{0}}\right)$$

where *n*_ID_ is the ideal factor, *k*_B_ is the Boltzmann constant, *J*_0_ is the dark current, it is clear that severe non‐radiative recombination will enhance the *J*_0_ significantly, therefore degrading the *V*_oc_ substantially. If the *J*_0_ is enhanced by two orders, *V*_oc_ will decrease by 0.118 V (assuming that *n*_ID_ is 1 and *J*_sc_ is 20 mA cm^−2^).

**Figure 3:**
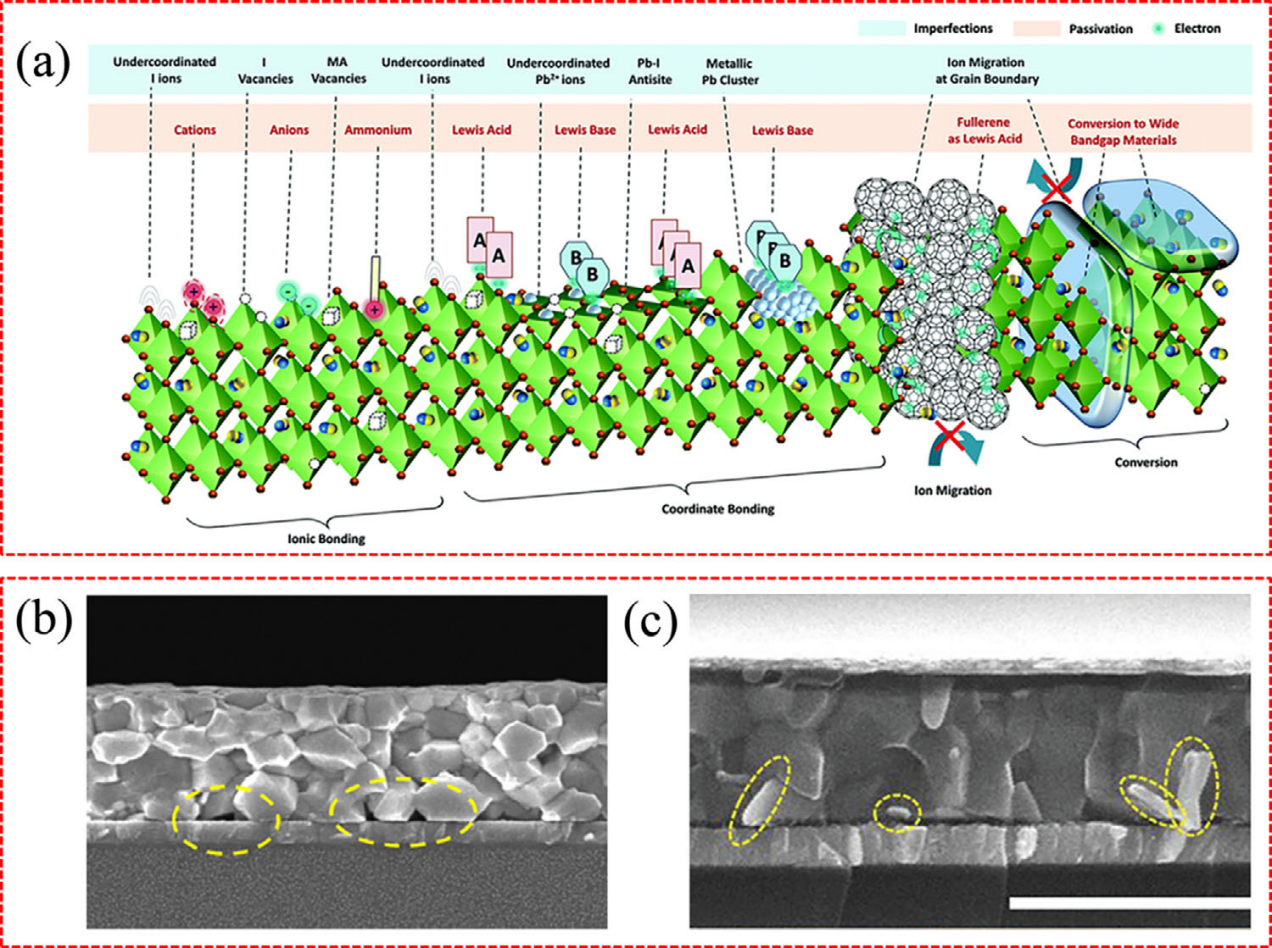
a) Imperfections in perovskite film and their passivation by ionic bonding, coordinate bonding, and conversion to wide bandgap materials, and suppression of ion migration at extended defects. Reproduced with permission.^[^ ^88^ ^]^ Copyright 2019, Royal Society of Chemistry. b) Cross‐sectional SEM image of the control perovskite film. The yellow circles show the interfacial voids with a depth of ≈100 nm. Reproduced with permission.^[^ ^89^ ^]^ Copyright 2021, American Association for the Advancement of Science. c) Cross‐sectional SEM image of inverted PSCs. The sub‐microscale imperfections are highlighted by the yellow dotted circles or ellipses. Reproduced with permission.^[^ ^90^ ^]^ Copyright 2021, Wiley‐VCH.

These defects can have a detrimental impact on the performance and stability of PSCs, as they can reduce the efficiency of charge transport and extraction, and increase the rate of non‐radiative recombination. **Figure** **3**a illustrates the primary defects found in perovskite films,^[^ ^88^ ^]^ including those that can give rise to deep‐level traps and shallow‐level traps. Typically, undercoordinated halide ions, undercoordinated Pb^2+^ ions, lead clusters (Pb^0^), and occasionally a few intrinsic point defects such as antisite defects, are identified as the significant sources of deep‐level traps, leading to non‐radiative recombination. In addition, morphological defects such as voids^[^ ^89^ ^]^ (Figure 3b) and flaky lead‐halide grains^[^ ^90^ ^]^ (Figure 3c) present at the bottom of perovskite films serve as deep‐level traps, severely undermining the performance and stability of the devices. Huang et al. proposed that the inefficient extraction of photogenerated holes by the HTLs in void‐rich regions triggers charge accumulation, a process that exacerbates perovskite degradation through heightened ion migration.^[^ ^89^ ^]^ Meanwhile, these voids function as storage sites for light‐induced decomposition byproducts, including iodine vapor species that form within the perovskite matrix during illumination and propagate self‐amplifying degradation pathways.

**Figure 4:**
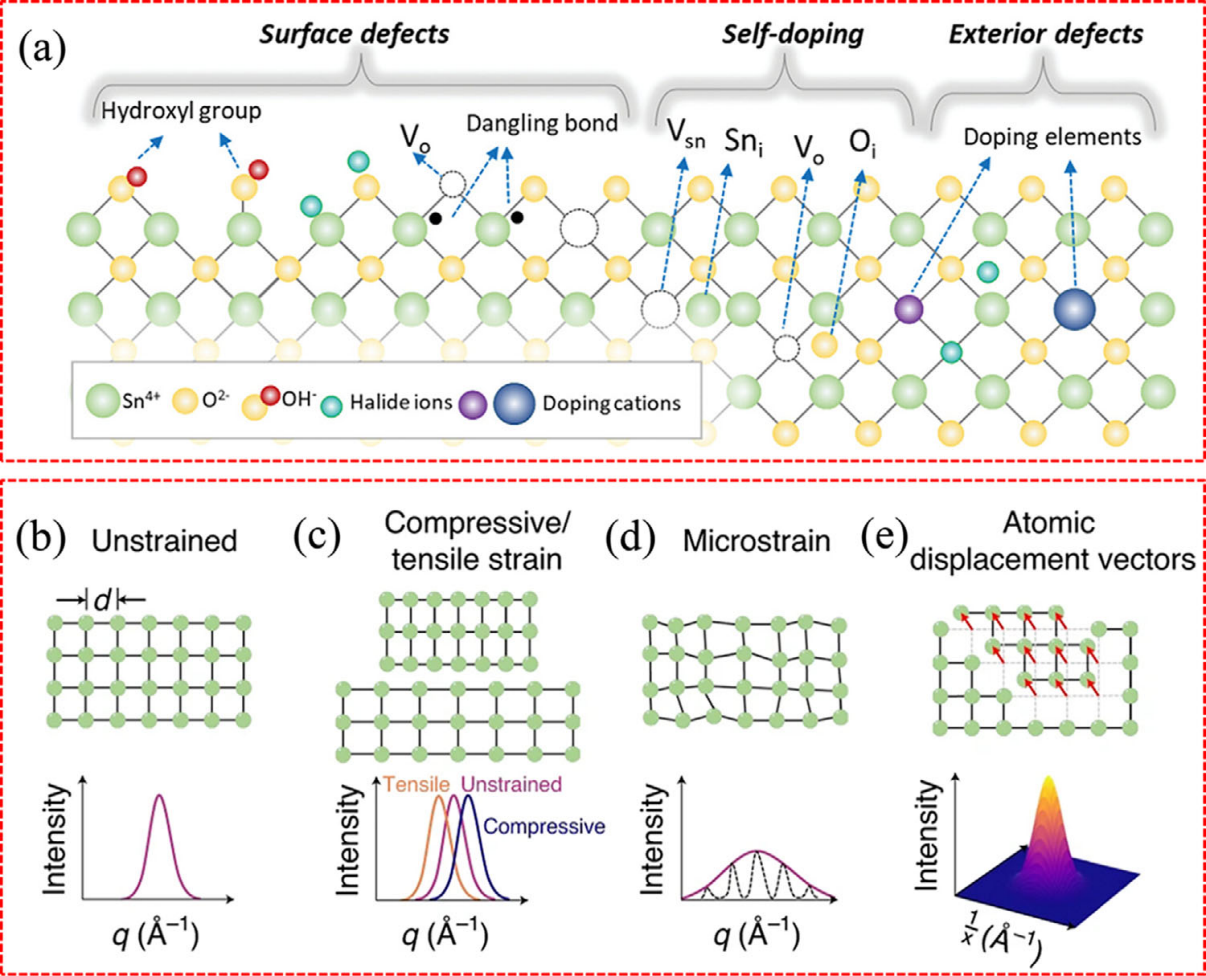
a) Schematic illustration of various possible defects in SnO_2_ surface and crystals. Reproduced with permission.^[^ ^59^ ^]^ Copyright 2022, Wiley‐VCH. The top row gives b) representations of unstrained material, c) compressive/tensile strain, d) microstrain, and e) atomic displacement vectors. The bottom row indicates the signatures of each of these kinds of strain in diffraction patterns. b–e) Reproduced with permission.^[^ ^99^ ^]^ Copyright 2021, Springer Nature.

As reported, metal oxide CTLs (e.g., SnO_2_, ZnO, TiO_2_, NiO_x_) are found to exhibit a significant presence of surface defects or trap states, including hydroxyl (OH) groups, oxygen vacancies (V_O_), and undercoordinated metal atoms.^[^ ^91^ ^]^ Taking SnO_2_ as an example, **Figure** **4**a illustrates the possible defects on the surface and inside the crystal of metal oxides.^[^ ^59^ ^]^ Jung et al. found that the OH groups present on the SnO_2_ surface can be classified into two types: terminal hydroxyls (OH_T_), which introduce deep energy states, and bridge hydroxyls (OH_B_), impacting the energy level.^[^ ^92^ ^]^ Research findings indicate that SnO_2_ surfaces undergo a significant generation of V_O_ during high‐temperature annealing. These V_O_ create shallow donor energy levels, resulting in n‐type conduction.^[^ ^93^ ^]^ However, the V_O_ can induce structural distortions of perovskite at the interface, forming undesired phases such as PbI_2_ and leading to severe recombination.^[^ ^94^ ^]^ It is noteworthy that the higher multivalency of Sn compared to Ti makes V_O_ more commonly observed on the surface of SnO_2_ than on TiO_2._^[^ ^95^ ^]^ Furthermore, it is known that SnO_2_ crystals have a significant number of Sn dangling bonds on their surface. These dangling bonds can absorb O_2_ and H_2_O from the atmosphere, trap electrons, and create potential barriers that impede electron transport.^[^ ^96^ ^]^

Indeed, the defects located at both the perovskite side and the CTL sides have a significant impact on device performance, specifically through the recombination process. It has been reported that in operating PSCs, the recombination process is predominantly influenced by deep‐level traps.^[^ ^88^ ^]^ This phenomenon can be described by the Shockley‐Read‐Hall (SRH) theory^[^ ^97^ ^]^: after the formation of deep‐level traps, they will only trap either electrons or holes, which are annihilated with an oppositely charged carrier through non‐radiative recombination rather than escape by thermal activation. SRH recombination has been identified as the primary pathway responsible for the loss of *V*_oc_. The magnitude of *V*_oc_ is determined by the gap between the quasi‐Fermi levels of electrons and holes. The external luminescence quantum efficiency (*η*_ext_) can be used to explain the relationship between the *V*_oc_ and non‐radiative recombination^[^ ^98^ ^]^:

(3) $$\;V_{\mathrm{OC}}=V_{\mathrm{OC},\;\mathrm{rad}}+\frac{kT}{q}\ln\left(\eta_{\mathrm{ext}}\right)$$

where *V*_oc, rad_ is the maximum achievable *V*_oc_ in the absence of any non‐radiative recombination processes. It can be deduced from this equation that the non‐radiative recombination process of excess free charge carriers diminishes the separation of quasi‐Fermi levels, resulting in a *V*_oc_ loss in PSCs.

### 3.3 Residual Strain at the Buried Interface

Residual strain at the buried interface exerts a significant impact on the optoelectronic properties of perovskite. Strain (*ε*) is the structural deformation that materials undergo in response to applied stress (*σ*_A_).^[^ ^99^ ^]^ It is commonly quantified as the ratio of the stress‐induced change in lattice parameters to the lattice parameters of the material in its stress‐free state (Figure 4b), and can be expressed as follows^[^ ^100^ ^]^:

(4) $$\varepsilon =\frac{\Delta a}{a_{0}}\;$$

Here, Δ*a* is the stress‐induced change in the lattice constant, and *a*_0_ represents the lattice constant of materials in their stress‐free state.^[^ ^101^ ^]^ Figure 4c illustrates that tensile strain arises when the lattice is stretched under stress, and compressive strain is generated by stress‐induced lattice contraction.^[^ ^102^ ^]^ Another notable aspect of strain is microstrain (Figure 4d, e), which is determined by analyzing the widths of Bragg peaks.^[^ ^99^ ^]^ In diffraction measurements, when multiple interplanar spacings (*d*) within a sample are simultaneously sampled, the combined contributions of these *d* spacings result in the formation of a widened Bragg peak. Microstrain acts as a metric for quantifying the local deviations of *d*‐spacing from its average value.

The strain observed at the buried interface of CTLs/substrate and perovskite is primarily caused by a mismatch in the coefficients of thermal expansion and lattice parameters between different materials. The crystallization process of perovskites entails a high‐temperature annealing treatment to remove residual solvents in the perovskite film. The subsequent cooling process can induce strain due to the mismatch in the coefficients of thermal expansion between the perovskite and the substrate.^[^ ^103^ ^]^ Typically, the substrate has a lower coefficient of thermal expansion than the perovskite layer, restricting the contraction of the perovskite. Consequently, this leads to in‐plane tensile strain while simultaneously causing out‐of‐plane compressive strain.^[^ ^104^ ^]^ Rolston et al. discovered a distinct linear correlation between the residual tensile strains in perovskite layers and the annealing temperature of the films.^[^ ^26^ ^]^ They observed that as the annealing temperature increased, the magnitude of the tensile strain also increased. To accurately quantify this relationship, they calculated the predicted strain (*ε*_Δ_ *_T_*) resulting from the thermal expansion mismatch using the equation:

(5) $$\varepsilon_{\Delta T}=\frac{E_{P}}{1-\upsilon_{P}}\;\left(\alpha_{s}-\alpha_{P}\right)\Delta T$$

Here, *E*_p_ denotes the modulus of the perovskite, *ν*_p_ represents the Poisson's ratio of the perovskite, *α*_s_ and *α*_p_ represent the respective coefficient of thermal expansions of the substrate and perovskite, respectively, and Δ*T* represents the temperature difference during the cooling process.

The lattice mismatch between the substrate and the perovskite can also give rise to strains in the as‐prepared perovskite layer. This strain is dependent on the difference in their respective lattice constants. The lattice mismatch (*δ*) can be quantified by the formula:

(6) $$\delta =\frac{a_{s}-a_{e}}{a_{s}}$$

where *a*_s_ is the lattice constant of the substrate, and *a*_e_ is the lattice constant of the perovskite. When *δ* is less than 9%, the perovskite grows into a strained layer in a pseudomorphic way to match the lattice constant of the substrate.^[^ ^100^ ^]^ However, if the *δ* value becomes relatively large, the perovskite crystals grow randomly on the substrate, leading to a disordered interface distortion.^[^ ^105^ ^]^ It is reported that the residual strain in the bottom region of the perovskite film is relatively higher than the regions farther away from the bottom,^[^ ^33^, ^106^ ^]^ indicating that the buried interface undergoes more severe lattice distortion.

The residual strain can significantly impact the optoelectronic properties of perovskite. Studies have reported that tensile strain enlarges the bandgap of perovskite, while compressive strain decreases the bandgap.^[^ ^23^ ^]^ Zhu et al. found that the presence of a tensile strain gradient in the perovskite film results in a rearrangement of energy levels and a decrease in hole mobility.^[^ ^107^ ^]^ It has been discovered that tensile strain lowers the activation energy for ion migration, leading to accelerated degradation of perovskite films.^[^ ^104^ ^]^ On the contrary, compressive strain raises the activation energy for ion migration, thereby contributing to the improvement of device stability.^[^ ^103^ ^]^ Saidaminov et al. reported that tensile strain decreases the formation energy of iodine vacancies in FAPbI_3_ films.^[^ ^108^ ^]^ These iodine vacancies exhibit a strong attraction to water and oxygen molecules, thereby leading to the degradation of perovskite films.

### 3.4 Carrier Dynamics at the Buried Interface

**Figure 5:**
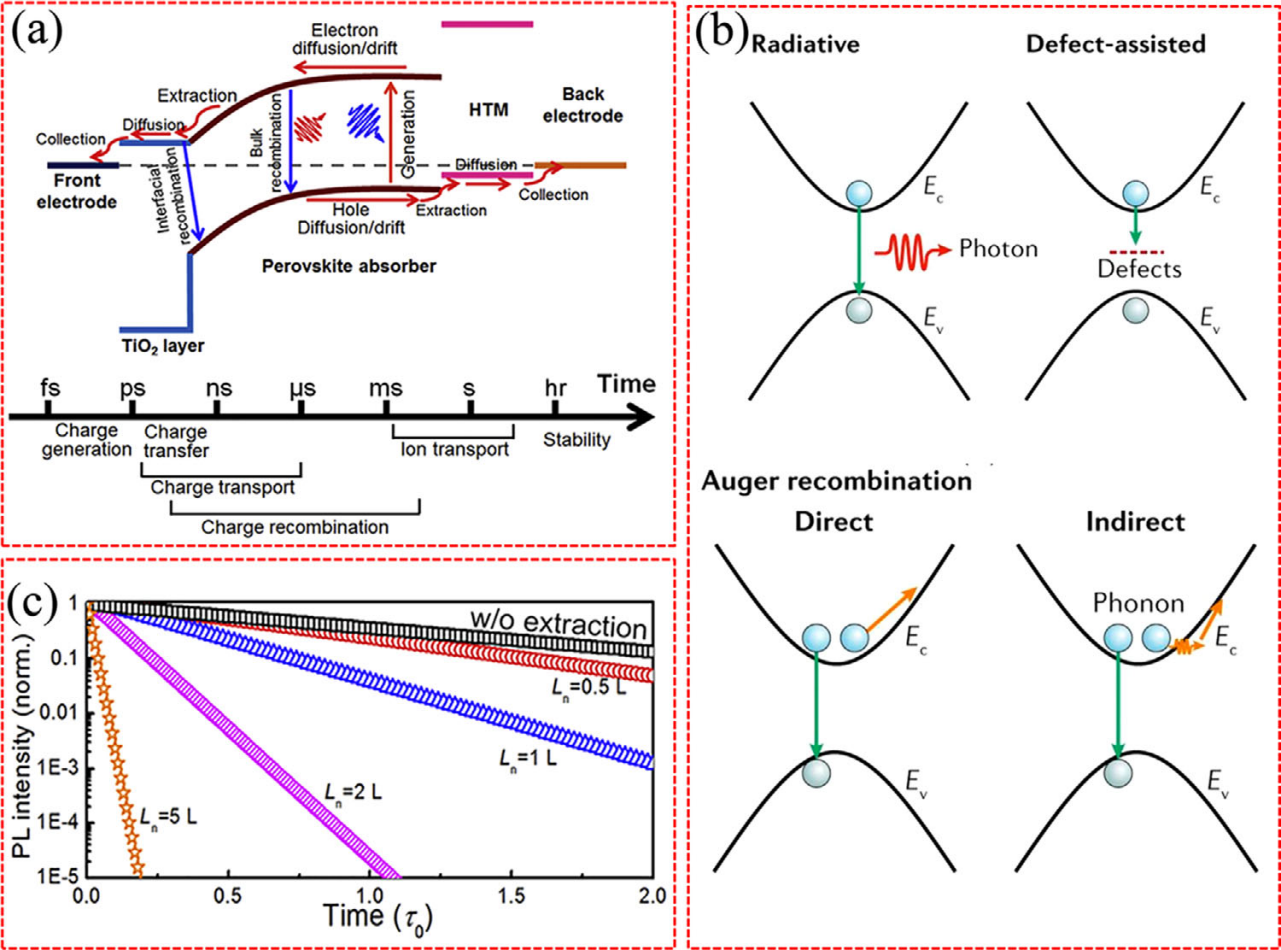
a) Possible charge processes and the corresponding timescales of the cell. b) Schematic diagram indicating recombination mechanisms active in organic–inorganic metal halide perovskites. Reproduced with permission.^[^ ^87^ ^]^ Copyright 2019, Springer Nature. c) Transient PL of the film with different carrier diffusion length (*L*_n_). a,c) Reproduced with permission.^[^ ^109^ ^]^ Copyright 2018, Elsevier.

The carrier dynamics at the buried interface also play a vital role in determining the performance of PSCs. Understanding the charge transport mechanisms, diffusion lengths, and recombination rates at the interface can provide valuable insights for designing the device architecture and choosing appropriate material composition. **Figure** **5**a illustrates the possible charge processes and corresponding timescales inside a PSC.^[^ ^109^ ^]^ The electrons in the valence band (VB) are excited to the conduction band (CB) by incident photons, which create electron–hole pairs called excitons. These excitons can be converted into photocurrent by separating them into free electrons and holes, overcoming the exciton binding energy. Perovskite has a low exciton binding energy of ≈16 meV,^[^ ^110^ ^]^ enabling spontaneous dissociation into free carriers at room temperature.^[^ ^111^, ^112^, ^113^ ^]^ Additionally, some carriers can be excited to higher energy levels or pumped to higher sub‐bands, which generates hot carriers. These hot carriers release their excessive energy through relaxation processes.^[^ ^114^ ^]^ Studies have demonstrated that excitons in perovskite are rapidly generated and dissociated into free charge carriers within picoseconds.^[^ ^115^ ^]^

Generally, the generated charge carriers are involved in two competing processes within the perovskite materials^[^ ^116^ ^]^: transport processes primarily driven by diffusion and drift mechanisms,^[^ ^117^, ^118^ ^]^ and recombination processes involving both radiative and non‐radiative recombination.^[^ ^119^ ^]^ The overall recombination process can be described as follows^[^ ^120^ ^]^:

(7) $$\frac{dn}{dt}=-k_{1}n-k_{2}n^{2}-k_{3}n^{3}$$

Here, k_1_, k_2_, and k_3_ respectively represent the first‐, second‐, and third‐order rate constants for defect‐assisted (monomolecular), radiative (bimolecular), and Auger (three‐body) recombinations, and n denotes the density of photon‐generated carriers.^[^ ^87^ ^]^ Shi et al. have reported that trap‐assisted non‐radiative recombination is the primary mechanism governing carrier decay under conditions of low photocarrier concentration (<10^15^ cm^−3^).^[^ ^109^ ^]^ In addition, the rate of defect‐assisted recombination is closely associated with the energy depth and density of defect states. As a consequence, reducing the presence of defects and surface states at the interface is expected to have a substantial impact on decreasing the first‐order recombination. Radiative recombination in perovskites constitutes the intrinsic bimolecular process where electrons and holes recombine across the band gap. This primarily occurs between the relaxed states—specifically, from the conduction band minimum (CBM) to the valence band maximum (VBM)—following the relaxation of carriers from higher energy states. The efficiency of this process is critically governed by momentum conservation between CBM and VBM, which is dynamically modulated by structural distortions in the perovskite lattice.^[^ ^87^, ^121^ ^]^ As shown in Figure 5b, Auger recombination is a higher‐order process that involves at least three particles, typically two electrons and a hole. In this process, the excess energy of an electron is transferred to another electron, facilitating non‐radiative recombination with a hole. In addition, Auger recombination strongly relies on the charge‐carrier density, which predominates in perovskite absorber layers where carrier concentrations exceed 10^17^ cm^−3.[^ ^122^, ^123^, ^124^ ^]^

In practical PSCs, in addition to undergoing bulk recombination, the free photocarriers can migrate through the perovskite film and be extracted into the CTLs. Typically, charge extraction takes place rapidly within a picosecond timeframe. Applying a high bias voltage weakens the built‐in electric field within the PSC, which significantly affects the distribution of charge carriers and the level of recombination losses. These dynamics are influenced by two crucial factors: the carrier diffusion length and the recombination lifetime. The carrier diffusion length (*L*_n_) is a parameter used to characterize the charge transport capability of the perovskite material. Transient PL is commonly used to evaluate the *L*_n_ of perovskite materials. Figure 5c demonstrates simulated PL decay curves with different *L*_n_ values, clearly indicating that a larger *L*_n_ corresponds to a shorter PL lifetime.^[^ ^109^ ^]^ When *L*_n_ is several times greater than the thickness of the perovskite film (*L*), the carrier extraction efficiency (*ƞ*_E_) can reach 100 percent. When *L*_n_ is comparable to *L*, *ƞ*_E_ is ≈80%. Therefore, enhancing the diffusion length and reducing the defect density in perovskite films are crucial for charge carrier extraction. In addition, optimal energy level alignment facilitates the establishment of a suitable built‐in electric field, which enhances effective charge transfer. It is widely acknowledged that an energy offset of ≈0.2 eV is necessary to ensure efficient charge extraction at the interfaces of CTLs/perovskite.^[^ ^122^ ^]^

Once the carriers are extracted, they undergo both horizontal and vertical diffusion within CTL before being collected by the electrodes. The dynamics of carriers within the CTL are mainly influenced by diffusion and interfacial recombination processes. Several research groups have also observed that the carrier transport time within the CTLs typically ranges in the order of microseconds.^[^ ^4^, ^125^, ^126^ ^]^ In order to mitigate detrimental effects such as electrode polarization and hysteresis resulting from charge accumulation at the interfaces, it is imperative to ensure efficient collection of charge carriers from the CTLs to the electrodes. It is worth noting that the efficiency of charge collection primarily depends on the charge transfer at the interfaces of CTLs/electrodes, as well as the conductivity of the electrode materials.^[^ ^116^ ^]^

### 3.5 Chemical Reactions at the Buried Interface

**Figure 6:**
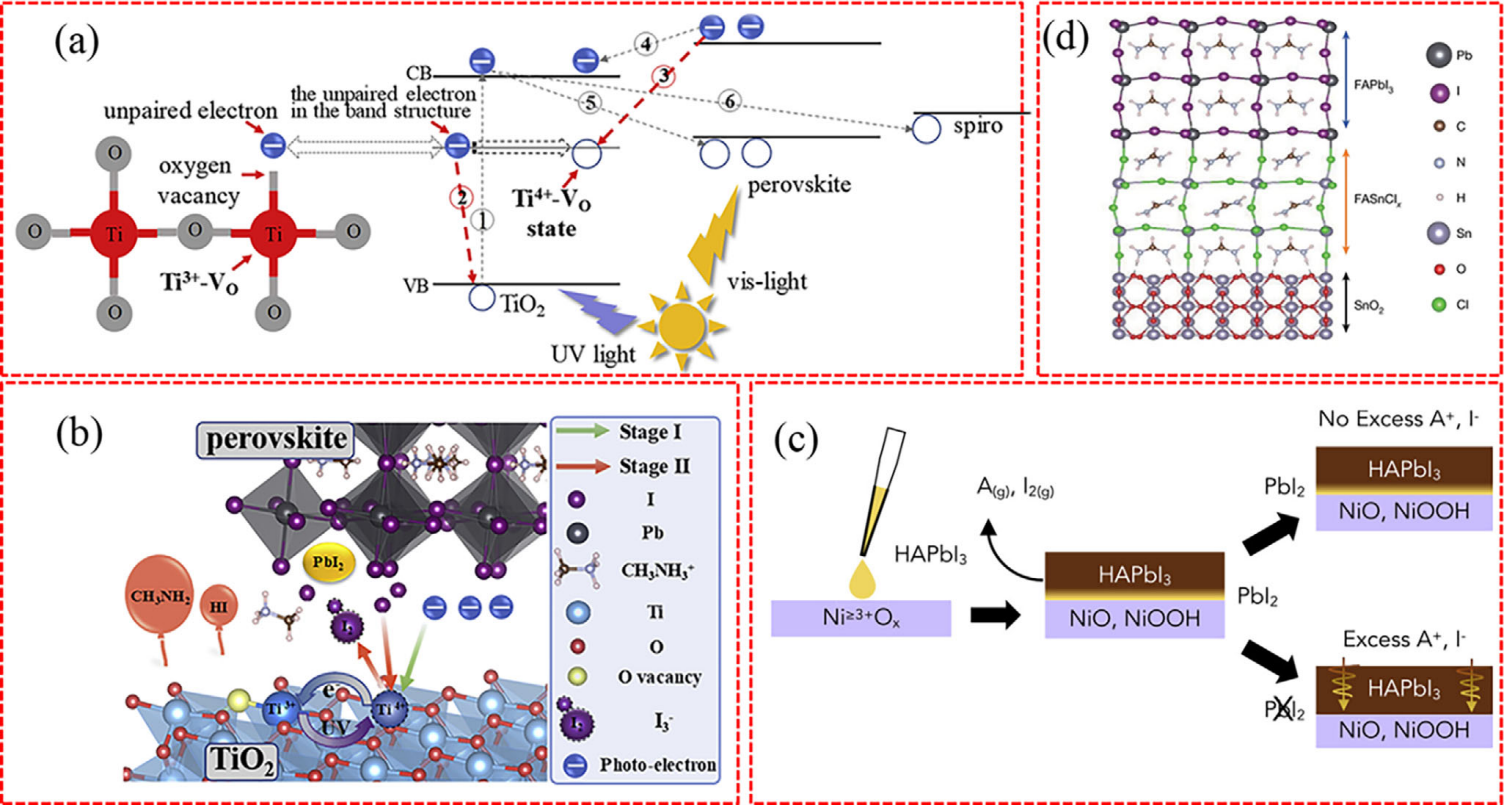
a) The energy band of TiO_2_ ETL and perovskite absorber. b) The mechanism of PSCs degradation under continuous UV irradiation. a,b) Reproduced with permission.^[^ ^131^ ^]^ Copyright 2020, Elsevier. c) Schematic of the addition of excess A‐site to the perovskite precursor, which titrates reactive sites at the NiO_x_ surface, preventing the formation of PbI_2‐x_Br_x_ at the interface. Reproduced with permission.^[^ ^137^ ^]^ Copyright 2020, Elsevier. d) Simulation of the formation of the interlayer between perovskite and SnO_2_. Reproduced with permission.^[^ ^34^ ^]^ Copyright 2021, Springer Nature.

Chemical reactions at the buried interface are of crucial importance for understanding the degradation mechanism of PSCs. The buried ETLs or HTLs, with their specific chemical properties, can initiate interactions with the adjacent perovskite layer. In most cases, these reactions exert an adverse influence on the quality and stability of the perovskite films. For example, in the preparation process, residual OH^−^ groups or acetate ligands might be present in the ZnO ETL.^[^ ^57^ ^]^ These ligands can react with CH_3_NH_3_^+^ ions, which disrupt the crystal structure of the perovskite. As a result, this process ultimately leads to the deterioration of the PSCs. The buried TiO_2_ ETL has been reported to catalyze the perovskite degradation under UV light soaking.^[^ ^46^, ^127^, ^128^, ^129^ ^]^ The driving force behind the decomposition process is likely to be attributed to the extraction of electrons from iodide ions by TiO_2_. Ito et al. proposed the mechanism of TiO_2_‐catalyzed decomposition of MAPbI_3._^[^ ^130^ ^]^ Initially, TiO_2_ can extract electrons from I^−^, leading to the formation of I_2_ and the subsequent disintegration of the perovskite crystal. In addition, CH_3_NH_3_^+^ ions will undergo rapid dehydrogenation with the assistance of H_2_O when exposed to humid air, releasing CH_3_NH_2_ gas. This step will cause an irreversible degradation of MAPbI_3_. Subsequently, H^+^ ions and I^−^ ions can undergo a reversible reaction to generate HI. Eventually, MAPbI_3_ completely decomposes into PbI_2_, HI, and CH_3_NH_2_. Ji et al. proposed a two‐stage degradation process in TiO_2_‐based PSCs.^[^ ^131^ ^]^ In the first decay stage, as shown in **Figure** **6**a,b, the presence of V_O_ combined with Ti^3+^ results in the formation of active Ti^4+^‐V_O_ defect states upon exposure to UV light, leading to the loss of photogenerated charge carriers. Additionally, the Ti^4+^‐V_O_ states can oxidize I^−^ back to Ti^3+^‐V_O_ states, which leads to the accumulation of I_3_^−^. Subsequently, the harmful accumulation of I_3_^−^ species accelerates the degradation of the perovskite, becoming the primary mechanism in the second stage of degradation.

The commonly used PEDOT:PSS HTL contains acidic components that can react with In_2_O_3_ and corrode indium tin oxide (ITO) glass. Dispersed In^3+^ ions can diffuse into the perovskite layer, which deteriorates the performance and stability of PSCs.^[^ ^69^, ^70^ ^]^ In addition, the acidic groups (e.g., sulfonic acid groups) in PEDOT:PSS can react with iodide ions in Sn–Pb perovskites, generating oxidizing species such as I_2_ or I_3_^−.[^ ^132^ ^]^ These species further undergo redox reactions with Sn^2+^, causing Sn^2+^ to lose electrons and form Sn^4+^, which exerts a detrimental impact on the performance of photovoltaic cells. The hygroscopic nature of PEDOT:PSS makes it vulnerable to moisture intrusion at the buried interface, leading to the absorption of moisture by the perovskite and, therefore the materials’ decomposition subsequently.^[^ ^133^, ^134^ ^]^ The interface between NiO_x_ and perovskite also undergoes chemical reaction processes. Extensive research has examined the chemical interactions between NiO_x_ HTLs and perovskites.^[^ ^135^, ^136^ ^]^ Boyd et al. has reported that Ni^≥3+^ ions selectively oxidize I^−^ ions due to the higher oxidation potential of Br^−^ and Cl^−^ ions.^[^ ^137^ ^]^ Simultaneously, CH_3_NH_3_^+^ ions undergo deprotonation reactions in the presence of Ni^≥3+^ ions. As illustrated in Figure 6c, the iodine‐based perovskites decompose at the buried NiO_x_/perovskite interface, resulting in the formation of CH_3_NH_2_ gas, I_2_, and PbI_2_. This formed PbI_2_ layer acts as a barrier, hindering the transport of holes to the NiO_x_ layer and ultimately reducing the overall performance of the device. They also observed that the presence of an excess of 1–5 mol% A‐site cations in the perovskite precursor solution inhibits the aforementioned decomposition reaction, resulting in an improved photovoltaic performance of the PSCs. As reported, the low oxidation activation energy of divalent Sn^2+^ ions in Sn‐based perovskite makes them prone to oxidation, resulting in the formation of tetravalent Sn^4+^ ions. Li et al. discovered that the undercoordinated Ni^≥3+^ ionic defects serve as Lewis acids and oxidants to oxidize the Sn^2+^‐based perovskite at the buried interface,^[^ ^138^ ^]^ which results in a significant reduction in the *V*_oc_ of Sn‐based PSCs.

In perovskite solar cells, ion migration under photo‐induced bias is closely coupled with interfacial chemical reactions,^[^ ^139^ ^]^ playing a critical role in device performance and stability. Photo‐generated carriers induce an internal electric field that drives directional migration of ions such as I^−^, Br^−^, and organic cations, which further trigger redox reactions. This ion migration‐reaction coupling can lead to lattice distortion of perovskites and accelerated chemical reactions with charge transport layers, ultimately affecting interfacial stability and device efficiency. For instance, Lin et al. found that ion migration can accelerate the reaction between oxygen and methylammonium lead iodide perovskite in light conditions.^[^ ^140^ ^]^ Specifically, under light, mobile ions (e.g., I^−^, iodine vacancies V_I_) in perovskites migrate under local electric fields, leading to the enrichment of V_I_ near electrodes. These V_I_ facilitate the formation of superoxide ions (O_2_^−^) from oxygen, which react with organic cations (e.g., MA^+^) to decompose the perovskite, generating more mobile ions and channels that further accelerate ion migration, creating a vicious cycle.

It is noteworthy that not all reactions occurring at the buried interface exert a detrimental effect on device performance. For example, the buried SnO_2_ containing Cl can react with FAPbI_3_ to form a FASnCl_x_ interlayer at the buried interface^[^ ^34^ ^]^ (see Figure 6d), which can effectively promote charge transport/extraction and reduce non‐radiative recombination. At the buried interface, Cl^−^ and I^−^ ions undergo spontaneous ion exchange. The diffusion of Cl^−^ ions into the perovskite layer facilitates the vertical growth of grains and diminishes defects at grain boundaries, which contributes to enhancing the crystalline quality of perovskite films. The aforementioned chemical reaction plays a crucial role in attaining unparalleled PCE and an exceptional operational stability in PSCs.

### 3.6 SAMs‐Related Issues

SAMs have currently been widely used in inverted PSC, valued for their ultrathin, ordered structure that enables low interfacial resistance, efficient hole extraction, and suppressed non‐radiative recombination. Their tunable molecular design also enhances compatibility with diverse perovskites. Yet, interface issues limit their application, as discussed below.

SAMs consist of one or a few layers of organic molecules that spontaneously adhere to solid substrate surfaces. The currently widely studied SAM molecules typically consist of three structural components: an anchoring group, a connecting group, and a terminal group.^[^ ^141^ ^]^ Various anchoring groups (─COOH, ─PO(OH)_2_, ─OH, ─SiR_2_OH, etc.) have been developed, which interact with substrates through different mechanisms‐primarily by reacting with surface ─OH groups or forming coordination bonds with metal atoms in the substrate, leading to distinct binding energies. The connecting group in the self‐assembled film serves as a molecular framework linking the anchoring and terminal groups. The terminal group of SAM molecules dictates the materials’ functional properties, surface characteristics, and interactions with overlying layers and the surrounding environment. Driven by the thermodynamic preference of the self‐assembly process, SAM molecules typically align vertically on the substrate.^[^ ^142^ ^]^ To date, SAMs have facilitated certified PCE exceeding 26.7%^[^ ^143^ ^]^ in single‐junction PSCs with regular bandgap, over 24%^[^ ^144^ ^]^ in wide‐bandgap single‐junction PSCs, beyond 29%^[^ ^145^ ^]^ in all‐perovskite tandem solar cells, and above 32%^[^ ^146^ ^]^ in P/S‐TSCs. The photovoltaic performance of recent SAMs‐based PSCs is summarized in **Table** **1**.

**Table 1:** The photovoltaic performance of recent SAMs‐based PSCs.

SAMs	Architecture	PCE [%]	Ref.
[(2‐(4‐(bis(4‐methoxyphenyl)amino) phenyl)‐1‐cyanovinyl)phosphonic acid (MPA‐CPA)	ITO/SAM/perovskite/Nd@C_82_/C60/BCP/Ag	26.78	[262]
4‐(benzothiophene) boronic acid (S‐BA)	ITO/NiO_x_/Mixed SAM/WBG perovskite/C60/SnO_2_/Au	20.10	[263]
2‐(3,6‐dimethoxycarbazol‐9‐yl)ethylphosphonic acid (MeO‐2PACz)	FTO/SAM/perovskite/C60/BCP/Ag	26.12	[264]
(4‐(7H‐dibenzo^[^c, g] carbazol‐7‐yl) butyl) phosphonic acid (4PADCB)	FTO/SAM/perovskite/C60/BCP/Cu	26.27	[265]
4‐(7H‐dibenzo[c,g]carbazol‐7‐yl)phenyl)phosphonic acid ( Bz‐PhpPACz)	FTO/SAM/perovskite/C60/BCP/Cu	26.39	[266]
(2‐(pyren‐1‐yl)ethyl)phosphonic acid (Py‐3)	ITO/SAM/perovskite/LiF/C60/BCP/Ag	26.10	[267]
[2‐(9H‐carbazol 9‐yl)ethyl]phosphonic acid (2PACz)	ITO/SAM/PyCA‐3F/perovskite/C60/BCP/Cu	25.12	[268]
[4‐(3,6‐dimethyl‐9H‐carbazol‐9‐yl) butyl]phosphonic acid (Me‐4PACz)	ITO/NiO/SAM/perovskite/PI/PCBM/BCP/Bi/Ag	26.54	[247]
(4‐(2,7‐dibromo‐9,9‐dimethylacridin‐10(9H)‐yl)butyl)phosphonic acid (DMAcPA)	ITO/perovskite/PC61BM/BCP/Ag	25.86	[269]
(4‐(3,6‐diphenyl‐9H‐carbazol‐9‐yl)butyl)phosphonic acid (Ph‐4PACz)	FTO/SAM/perovskite/C60/BCP/Ag	25.20	[270]
(4‐(3,11‐dimethoxy‐7H‐dibenzo[c,g]carbazol‐7‐yl)butyl)phosphonic acid (MeO‐4PADBC)	ITO/SAM/perovskite/2D passivation layer/C60/BCP/Ag	25.60	[227]
4‐(7‐(4‐(bis(4‐methoxyphenyl)amino)‐2,5‐difluorophenyl)benzo[c][1,2,5]thiadiazol‐4‐yl) benzoic acid (2F)	ITO/2F/WBG perovskite (1.77 eV)/C60/SnO_2_/Cu	19.33	[250]
(4 (9'‐phenyl‐9H,9'H‐[3,3'‐bicarbazol]‐9‐yl) butyl) phosphonic acid (4PABCz)	FTO/SAM/perovskite/PC61BM/BCP/Ag	26.90	[271]
[4‐(3,6‐dimethyl‐9H carbazol‐9‐yl)phenyl] phosphonic acid (Me‐PhpPACz)	ITO/SAM/perovskite/C60/BCP/Cu	26.17	[272]
(4‐(3,6‐dimethoxy‐9H‐carbazol‐9‐yl)phenyl)phosphonic acid (MeO‐PhPACz)	ITO/SAM/WBG perovskite (1.68 eV)/C60/BCP/Cu	21.10	[273]
Poly‐[2‐(9H‐carbazol 9‐yl)ethyl]phosphonic acid (Poly‐2PACz)	ITO/SAM/perovskite/C60/BCP/Cu	26.00	[274]

Although SAMs have emerged as promising hole transport materials in PSCs, but their application is hindered by critical interface‐related issues. One critical challenge is inadequate interface compatibility, where SAMs poorly match microstructured substrates (e.g., ITO/FTO, NiO_x_), leading to incomplete coverage and aggregation.^[^ ^147^ ^]^ This arises from two factors: binding energy heterogeneity‐anchoring groups (e.g., ─COOH, ─PO(OH)_2_) show variable reactivity with substrate ─OH groups or metal atoms, causing uneven adhesion and delamination, and topographical mismatch, where nanoscale textures (e.g., pyramids) disrupt film uniformity, leaving uncovered recombination centers. In addition, wettability and crystallization defects at perovskite‐SAM interfaces also pose significant hurdles: perovskite precursor solutions often exhibit poor wettability on SAM surfaces due to incompatible hydrophilic or hydrophobic properties between SAM terminal groups and solvents like DMF or DMSO, causing precursor droplet aggregation and resulting in rough or patchy films. Additionally, residual acidic anchoring groups like phosphonic acids interfere with perovskite nucleation, leading to uneven grain sizes and increased grain boundary defects.

### 3.7 Buried Interface Issues in P/S‐TSCs

In P/S‐TSCs, the buried interface between the perovskite layer and the underlying layers poses several critical issues. One of the primary concerns stems from the presence of micrometer‐sized pyramidal‐textured silicon. The textured silicon structure not only significantly influences the overlying HTLs but also further affects perovskite growth and morphology. Take NiO_x_ as an example: although it can uniformly coat textured substrates, it often contains numerous defects that cause interfacial degradation with the perovskite layer, thereby compromising device performance and stability.^[^ ^148^ ^]^ PTTA is a commonly used high‐conductivity layer in single‐junction PSCs. However, its application in tandem solar cells remains limited due to poor compatibility with textured substrates, which hinders efficient hole transport. Additionally, the hydrophobic aromatic rings in PTTA reduce the wettability of organic lead iodide inks, complicating film formation.^[^ ^40^ ^]^ Thermal evaporation of spiro‐thienopyridine (spiro‐TTB) is an alternative, but this material tends to aggregate in the pyramid valleys of ITO‐coated silicon‐based cells, leading to uneven layer deposition.^[^ ^149^ ^]^ SAMs are typically anchored on ITO, can address some of these interfacial defects. Nevertheless, the poor wetting of subsequent organic lead iodide precursor inks on SAMs‐modified surfaces threatens device reproducibility and scalability.^[^ ^22^ ^]^

Moreover, the interaction between the perovskite and the textured silicon substrate at the buried interface is complex. The textured silicon structure can influence the growth and morphology of the perovskite layer in ways that are not fully understood. The topography of the silicon texture can cause differences in the local environment for perovskite crystallization. In some cases, the perovskite crystals may grow preferentially in certain directions or regions, which can lead to an anisotropic distribution of charge transport properties within the perovskite layer. This anisotropy can further complicate the charge extraction and collection processes, ultimately affecting the performance of the P/S‐TSCs. While additive engineering,^[^ ^150^ ^]^ spray coating,^[^ ^151^ ^]^ or thermal evaporation strategies may offer partial solutions, they inevitably complicate the manufacturing process and introduce compatibility challenges.

## 4 Strategies for the Buried Interface Issues

### 4.1 Defects Passivation

**Figure 7:**
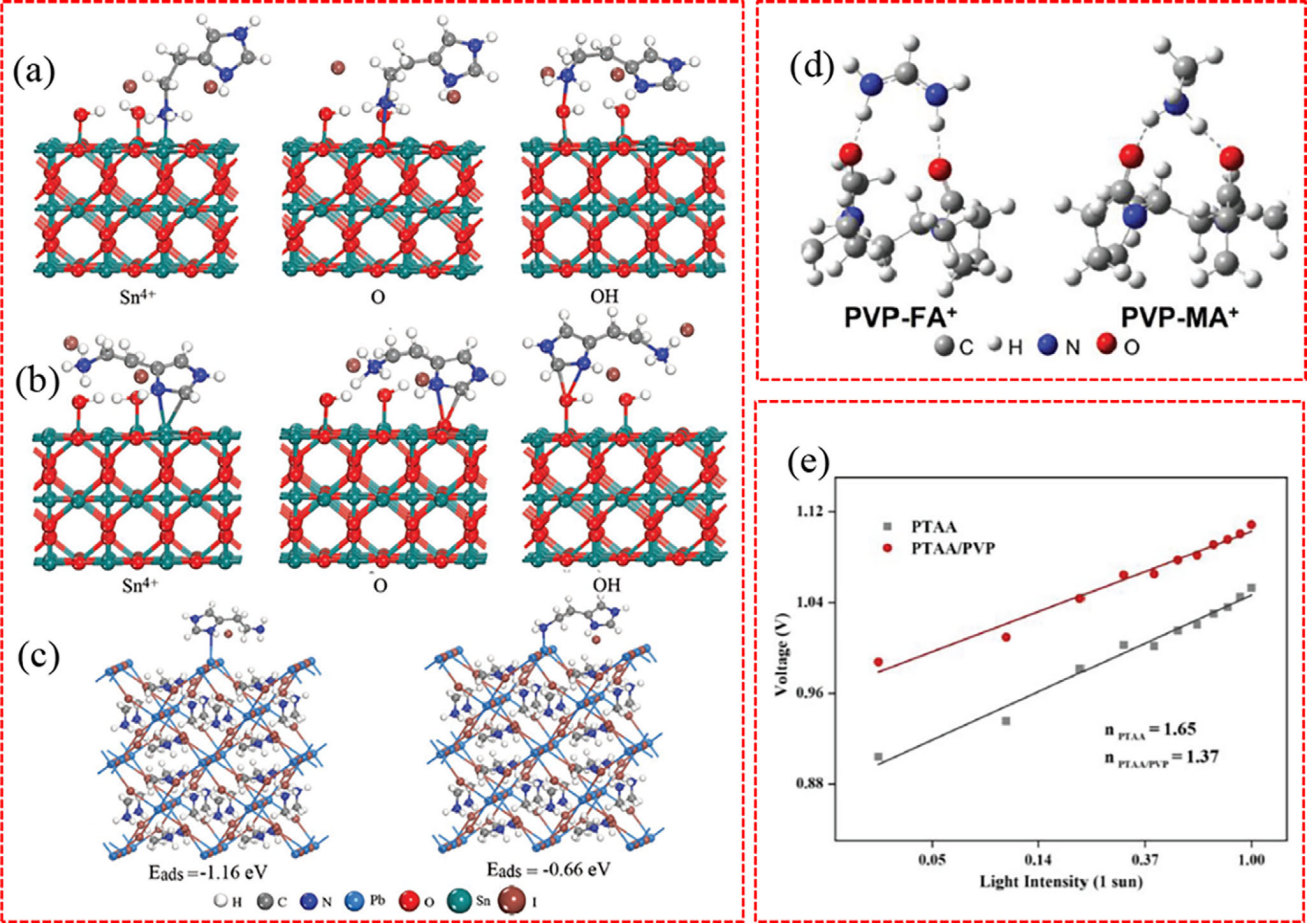
a) Adsorption geometries of N in tail ─NH_3_ of HADI at Sn^4+^, reactive oxygen species, and hydroxyl groups on the SnO_2_ surface, respectively. b) Adsorption geometries of C═N in the imidazole ring of HADI at Sn^4+^, reactive oxygen species, and hydroxyl groups on the SnO_2_ surface, respectively. c) Adsorption geometries of C═N in the imidazole ring or N in the tail ─NH_3_ of HADI at Pb^2+^ on the perovskite surface. a–c) Reproduced with permission.^[^ ^155^ ^]^ Copyright 2022, Wiley‐VCH. d) The optimized geometries of PVP‐FA^+^ and PVP‐MA^+^, and the calculated interaction energies are ‐34.5 or −26.8 kcal mol^−1^, respectively. e) Light intensity‐dependent *V*_oc_ plots. d,e) Reproduced with permission.^[^ ^261^ ^]^ Copyright 2024, Wiley‐VCH.

The existence of defects at the buried interface presents substantial challenges to device performance and stability. Interfacial defects can result in elevated rates of non‐radiative recombination, reduced efficiency of charge transport and extraction, and the accumulation of charges at the interface. Consequently, it becomes crucial to mitigate these defects at the buried interface to improve the performance and stability of PSCs. Organic materials are commonly used to passivate defects and improve the quality of perovskite films due to their changeable and various functional groups, which can strongly interact with certain types of defects at the buried interface.^[^ ^22^, ^152^, ^153^, ^154^ ^]^ The coordination bonds formed between functional groups containing heteroatoms such as O, N, and S and undercoordinated Pb^2+^ typically have bond energies ranging from 10 to 30 kcal/mol,^[^ ^155^ ^]^ which is significantly higher than the electrostatic interactions between inorganic ions (5–15 kcal mol^−1^). This strong covalent interaction can stably occupy defect sites and reduce the probability of desorption under thermal disturbances. For example, as shown in **Figure** **7**a–c, histamine diiodate (HADI) is recognized as a chemical bridge between SnO_2_ and perovskite.^[^ ^155^ ^]^ In this configuration, the imidazole unit of HADI shows a preference for binding with undercoordinated Pb^2+^ ions, while the ─NH_3_ group interacts with ─OH groups on the surface of SnO_2_. This bridging interaction significantly reduces the amount of defects at the buried interface. Some organic materials can form cyclic intermediates by hydrogen bonding with cations (FA^+^, MA^+^) in perovskites, regulating crystallization kinetics, delaying the crystallization process to reduce defect density, thereby effectively inhibiting non‐radiative recombination. For instance, the interlayer polymer polyvinyl pyrrolidone (PVP) undergoes spontaneous hydrogen bonding interactions with FA^+^ or MA^+^ in the perovskite^[^ ^156^ ^]^ (see Figure 7d), leading to the formation of a cyclic intermediate. This intermediate serves as an obstacle to the formation of other intermediates and slows down the crystallization process of the perovskite. Consequently, perovskite with reduced defect films is achieved, and non‐radiative recombination at the buried interface is effectively suppressed (Figure 7e). Other organic materials, such as poly(ethylene glycol) diacrylate,^[^ ^157^ ^]^ ionic liquid crystal (ILC, 1‐Dodecyl‐3‐methylimidazolium tetrafluoroborate),^[^ ^158^ ^]^ bathocuproine (BCP),^[^ ^159^ ^]^ 3‐aminopropanoic acid,^[^ ^160^ ^]^ 5‐aminovaleric acid (5‐AVA),^[^ ^161^, ^162^ ^]^ EDTA,^[^ ^163^, ^164^ ^]^ heparin potassium (HP),^[^ ^165^ ^]^ pentylammonium acetate (PenAAc),^[^ ^166^ ^]^ ammonium sulfamate (AS),^[^ ^167^ ^]^ and so on, have also been reported to be used for passivating defects at the buried interface, thereby enhancing the photovoltaic performance and stability of devices. In those cases, the active groups or atoms (O, N, S) act as passivating centers to interact with the defects, typically through coordination bonds.

**Figure 8:**
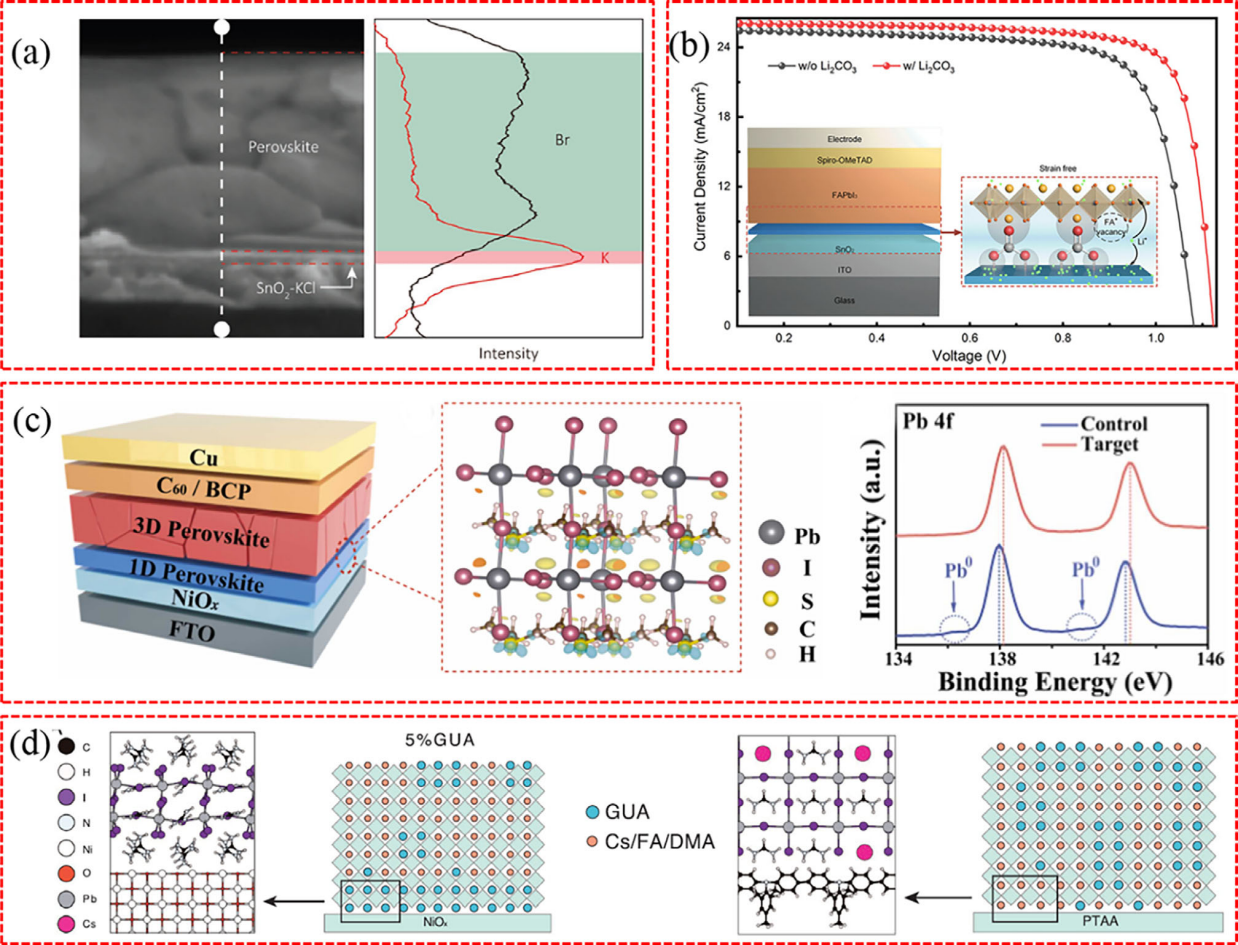
a) Elemental scanning in a linear mode through the cross‐section of SnO_2_ KCl/perovskite. The white dashed line in the figure indicates the linear scanning direction. Reproduced with permission.^[^ ^168^ ^]^ Copyright 2019, Wiley‐VCH. b) Defect passivation mechanism of Li_2_CO_3_. Reproduced with permission.^[^ ^18^ ^]^ Copyright 2022, American Chemical Society. c) Left: device architecture; Middle: differential charge density simulation of 1D interlayer with (CH_3_)_3_SI and PbI_2_ (Blue and yellow mean losing and gaining electrons, respectively); Right: XPS spectra of Pb 4 f for perovskite films. Reproduced with permission.^[^ ^178^ ^]^ Copyright 2024, Elsevier. d) Mechanism of substrate‐induced preferential crystallization of GUA_2_PbI_4_. Structural model and corresponding schematics of crystallization process for films with 5% GUA concentration on NiO_x_ (left) and PTAA (right) substrate. Reproduced with permission.^[^ ^176^ ^]^ Copyright 2021, Wiley‐VCH.

Inorganic materials are also an effective class of defect passivators.^[^ ^168^, ^169^, ^170^, ^171^, ^172^ ^]^ Inorganic passivators interact with perovskites primarily through ionic bonds and electrostatic interactions, differing fundamentally from organic materials that rely on molecular‐level coordinate bonding. Their short‐range electrostatic effects, driven by charge attraction between ions (e.g., K^+^, Cl^−^), focus on charge compensation (including filling halide vacancies or neutralizing surface defects) without the directional specificity of organic ligands. Inorganic ions often diffuse into perovskite lattices, modifying crystal parameters (e.g., Cs^+^ reducing cell volume) while passivating defects. This dual “doping‐repair” action contrasts with organic materials’ limited influence within a few surface layers. For instance, Zhu et al. discovered that introducing KCl into the SnO_2_ ETL not only passivates defects at the buried interface but also passivates the grain boundaries within the bulk perovskite. As presented in **Figure** **8**a, this is attributed to the diffusion of K^+^ ions into the perovskite.^[^ ^168^ ^]^ In addition, other K^+^‐containing salts, such as KSCN,^[^ ^172^ ^]^ KOH,^[^ ^173^ ^]^ and KAc,^[^ ^172^ ^]^ can also have a passivation effect on defects at the buried interface. Zhuang et al. found that rubidium fluoride (RbF) interlayer effectively passivates interstitial halogen (I_i_ and Br_i_) defects at the SnO_2_/perovskite interface and grain boundaries.^[^ ^174^ ^]^ As shown in Figure 8b, Li_2_CO_3_ effectively reduces the V_FA_ vacancies at the SnO_2_/FAPbI_3_ interface due to the strong interaction of CO_3_^2−^ with FA^+^ and SnO_2._^[^ ^18^ ^]^ Liu and his coworkers also discovered that the NH_4_Cl modification layer can effectively reduce the density of deep‐level antisite defects at the buried interface. This reduction can be attributed to two factors: the higher formation energy of Pb–Cl antisites compared with Pb–I antisites and the stronger binding between the perovskite and SnO_2_ (110) surface.^[^ ^175^ ^]^

In addition to the modification of CTLs (bulk or surface), some reports have revealed that additives in perovskite precursor solutions can interact with CTLs to form buried perovskite, thus passivating defects at the buried interface.^[^ ^34^, ^176^ ^]^ For example, the addition of choline acetate (ChA) has been shown to react with PbI_2_, forming 1D ChPbI_3_ perovskite at the buried interface.^[^ ^177^ ^]^ This interaction effectively reduces interfacial defects and prolongs the carrier lifetime. In Figure 8c, the newly formed 1D (CH_3_)_3_SPbI_3_ perovskite at NiO_x_/perovskite interface can effectively mitigate Pb^0^ defects due to the strong bonding between Pb and S.^[^ ^178^ ^]^ Chen et al. found that using a precursor solution containing GUA can result in the formation of a buried 2D GUA_2_PbI_4_ perovskite phase,^[^ ^176^ ^]^ which is mainly due to the symmetrical structure of GUA and its abundance of ─NH_2_ functional groups. As shown in Figure 8d, the hydrogen on GUA's amino groups interacts with the undercoordinated oxygen on the NiO_x_ surface, providing favorable sites for the formation of 2D GUA_2_PbI_4_. The newly formed buried perovskite effectively passivates the defects on the NiO_x_ surface, thereby reducing non‐radiative recombination at the NiO_x_/perovskite interface.

It is worth noting that some materials with special structures, such as low‐dimensional materials, metal‐organic frameworks (MOFs), carbon materials, and SAMs, can also serve as eligible passivators for perovskite devices. For example, 0D MoS_2_ quantum dots (QDs) have been reported to successfully reduce defects at the buried interface of PTAA/perovskite, which is proven to extend the lifetime of carriers and reduce carrier recombination.^[^ ^179^ ^]^ 2D black phosphorene (BP) distributed on the TiO_2_ surface is reported to act as heterogeneous nucleation centers during the perovskite crystallization process.^[^ ^180^ ^]^ This material reduces the nucleation density of the perovskite crystal, thereby promoting the growth of larger grains. Consequently, the resulting perovskite thin films exhibit reduced grain boundaries and a significantly lower density of defects. Dou et al. innovatively integrated Eu‐MOF modification layer on SnO_2_ surface, enabling the utilization of Eu^3+^‐Eu^2+^ redox ion pairs to convert I^0^ and Pb^0^ defects into I^−^ and Pb^2+^, effectively eliminating these defects.^[^ ^181^ ^]^ Furthermore, the organic ligands in the Eu‐MOF contain N atoms that can strongly interact with undercoordinated Pb^2+^ defects, leading to the efficient passivation of exposed Pb^2+^. Moreover, fullerene‐based derivatives have also been employed in buried interface modification.^[^ ^182^, ^183^, ^184^ ^]^ For example, Zhang et al. employed a C_60_ derivative,[6, 6]‐4‐fluorophenyl‐C_61_‐butyric acid (FPAC_60_), as a SAM on the surface of SnO_2_.^[^ ^185^ ^]^ By acting as a nucleation site, the FPAC_60_ SAM controlled the crystallization process of perovskite films and effectively mitigated defects at the buried interface. Recently, SAMs have been commonly used to passivate defects on the surface of CTLs.^[^ ^186^, ^187^, ^188^ ^]^ Zhou et al. discovered that incorporating a TBT‐BA SAM interlayer between NiO_x_ and perovskite,^[^ ^188^ ^]^ which consists of methoxy‐substituted triphenylamine‐functionalized benzothiadiazole (TBT) and benzoic acid (BA), enhances the binding energy with perovskite. This enhanced interaction effectively reduces the buried defects, making the passivation strategy highly effective.

### 4.2 Strain Control

**Figure 9:**
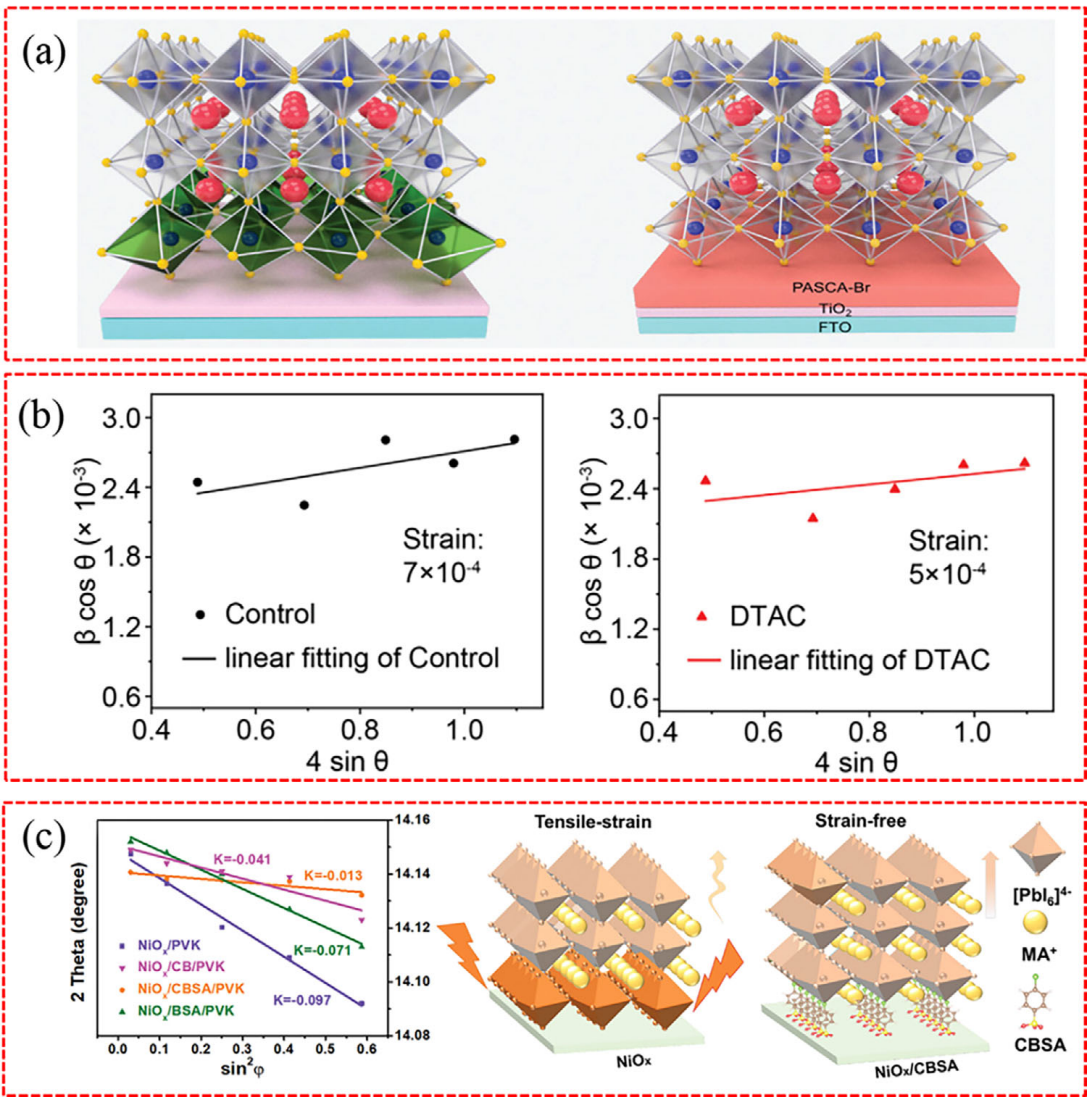
a) A schematic of perovskite crystals at the interface before (left) and after (right) PASCA‐Br modification. Reproduced with permission.^[^ ^105^ ^]^ Copyright 2020, Wiley‐VCH. b) The lattice strains of perovskite films deposited on PTAA and DTAC were calculated as the slope of the linear fitting. Reproduced with permission.^[^ ^193^ ^]^ Copyright 2022, Wiley‐VCH. c) The residual stress distribution (2θ data as a function of sin2φ) of perovskite films deposited on NiO_x_, NiO_x_/CB, NiO_x_/CBSA, and NiO_x_/BSA at different tilt angles (left). Schematic diagrams of lattice strain distribution in perovskite grains deposited on NiO_x_ and NiO_x_/CBSA substrates (right). Reproduced with permission.^[^ ^201^ ^]^ Copyright 2022, Wiley‐VCH.

Typically, residual tensile strain distorts PbI_6_ octahedra through weakening Pb─I bonds, leading to a widened band gap because of the antibonding nature of the valence band edge.^[^ ^189^ ^]^ In addition, this strain leads to the formation of defects and facilitates ion migration. Therefore, precise control of lattice strain is essential for improving photovoltaic performance and stability of PSCs. By controlling the lattice strain and promoting efficient carrier transfer, these strategies can enhance the performance and stability of PSCs. In this context, the main focus is on controlling lattice strain through modifications in the buried interface. One strategy to solve this problem is to insert flexible interlayers at the buried interface to release the lattice strain.^[^ ^105^, ^190^, ^191^ ^]^ For instance, Wu et al. introduced polystyrene (PS) at the buried interface between SnO_2_ and the perovskite.^[^ ^190^ ^]^ The low glass transition temperature of PS enables the perovskite to release lattice strain during the crystallization and annealing processes, resulting in strain‐free perovskite films with larger lattice structures. Zhang et al. also discovered that the flexible interlayer of the protonated amine silane coupling agent (PASCA‐Br) can establish a firm connection between TiO_2_ and perovskite by securely bonding both materials.^[^ ^105^ ^]^ The alkyl ammonium bromide terminals (R‐NH_3_Br) serve as structural components within the lattice unit of the distorted octahedra in the perovskite crystals. As shown in **Figure** **9**a, the presence of stretchable growth sites provided by PASCA‐Br enables the perovskite to release lattice strain. As a result, interfacial strain is reduced, leading to improved device stability.

Adjusting the mismatches in thermal expansion coefficients between the substrate and the perovskite is another strategy to reduce lattice strain.^[^ ^192^ ^]^ Lv et al. discovered that the dodecyltrimethylammonium chloride (DTAC) interlayer exhibits compression or stretching behavior, effectively facilitating self‐adaptation to the contraction mismatch between the perovskite and substrate.^[^ ^193^ ^]^ As shown in Figure 9b, the presence of DTAC successfully suppressed lattice distortion and released interfacial residual strain during perovskite crystallization and thermal annealing. A buffer layer of piperazine dihydriodide (PDI_2_) is also inserted at the buried interface between PTAA and the perovskite to control the strain.^[^ ^191^ ^]^ This PDI_2_ layer enhances the wetting of the PTAA substrate and induces an increased surface energy. Furthermore, the flexible nature of PDI_2_ makes it self‐adapt to the mismatch in thermal expansion coefficients between PTAA and the perovskite, thus releasing lattice strain and resulting in a defect‐less buried interface. As a result, the efficiency and operational stability of the PSCs are improved.

The residual strain can also be released by lattice matching at the buried interface, which means employing CTLs with a lattice constant comparable to that of perovskite can control the strain.^[^ ^194^, ^195^, ^196^, ^197^, ^198^ ^]^ Zhou et al. reported that the interlayer of WS_2_ nanoflakes facilitates the epitaxial growth of CsPbBr_3_ crystal due to their lattice structure compatibility,^[^ ^106^ ^]^ which resulted in the formation of a WS_2_/CsPbBr_3_ van der Waals heterostructure interface. This interface between ETL and CsPbBr_3_ assists in lattice expansion or shrinkage, thereby releasing interfacial tensile strain. Along these lines, Zhao's group designed a transparent conductive oxide perovskite (SrSnO_3_) to serve as ETL.^[^ ^199^ ^]^ Cs_0.05_(FAMA)_0.95_PbI_3_ is epitaxially grown on this ETL as a template, allowing for more orderly crystalline growth due to the matched lattice structure between Cs_0.05_(FAMA)_0.95_PbI_3_ and the SrSnO_3_ substrate. This process results in a defect‐less and strain‐free perovskite film, which facilitates efficient carrier transport and reduces interface non‐radiative recombination at the buried interface. As a consequence, the device achieves an impressive efficiency of 25.17%. A novel HTL, LaNiO_3_, has been reported as a promising alternative to conventional NiO_x_ HTL for stable p–i–n PSCs.^[^ ^200^ ^]^ The lattice constant of LaNiO_3_ closely matches that of MAPbI_3_, resulting in minimal residual strain within the perovskite film. This excellent compatibility between LaNiO_3_ and MAPbI_3_ contributes to improved quality of the perovskite film, enhancing the overall performance and stability of PSCs. A self‐assembled small‐molecule (SASM) interlayer known as p‐chlorobenzenesulfonic acid (CBSA) has been developed as a growth inducement platform for perovskite.^[^ ^201^ ^]^ As illustrated in Figure 9c, a smaller slope indicates less residual strain in the perovskite. It is evident that the presence of CBSA can eliminate interfacial lattice mismatch by regulating the oriented growth of perovskite crystals, ultimately resulting in the production of a high‐quality perovskite film. It is noteworthy that in multilayer devices such as all‐perovskite and P/S‐TSCs, lattice matching is also feasible by optimizing lattice parameters of each functional layer to achieve cross‐interface strain synergistic regulation, avoiding strain accumulation. This is supported by examples like ITO/IZO interlayers matching both perovskite and silicon lattices.^[^ ^76^, ^202^ ^]^

### 4.3 Carrier Transport Regulation

**Figure 10:**
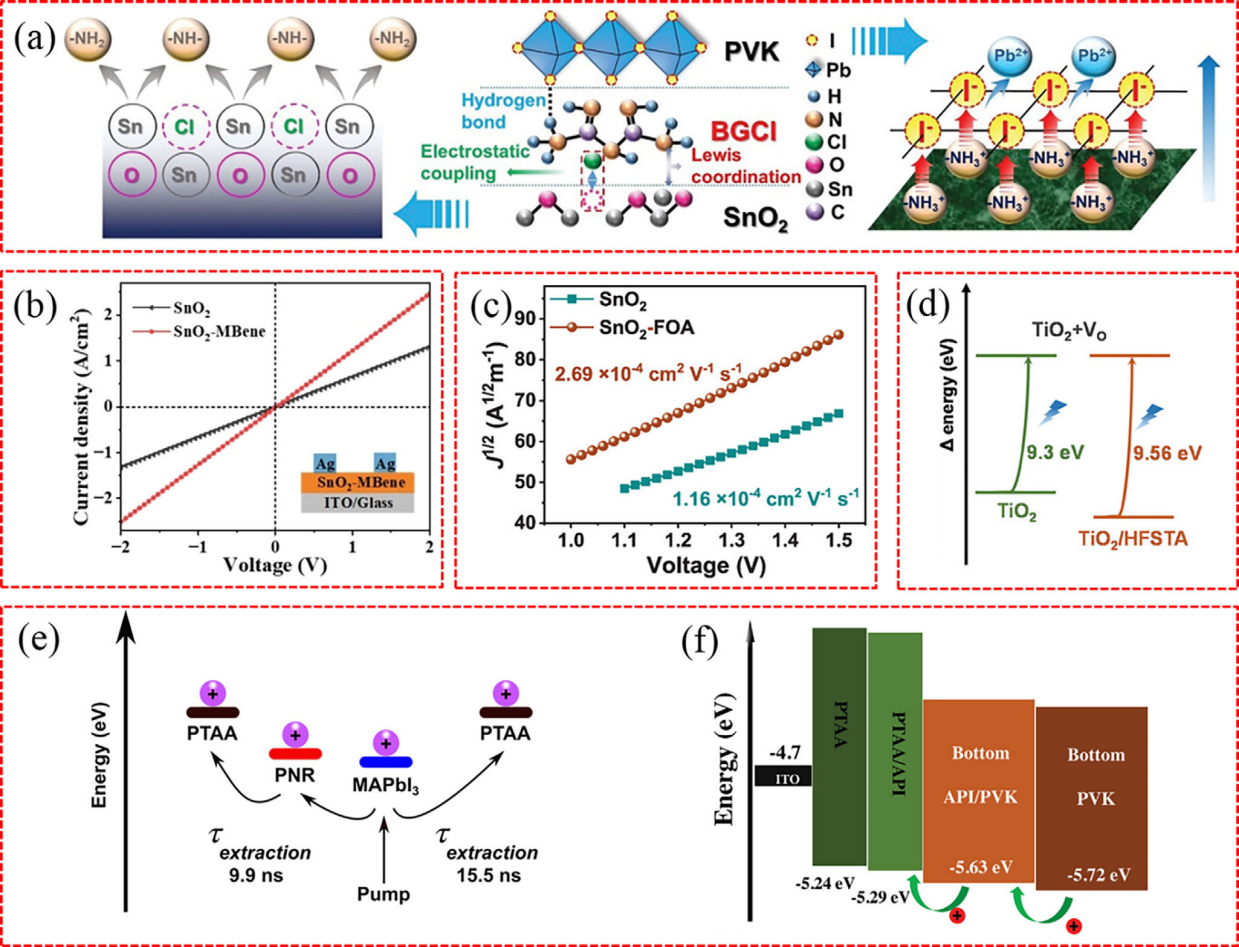
a) Schematic of the proposed modification mechanism of the BGCl at the ETL/PVK interface. Reproduced with permission.^[^ ^203^ ^]^ Copyright 2021, Wiley‐VCH. b) *I*–*V* characteristics of the ITO/SnO_2_ or SnO_2_‐MBene/Ag device in the dark. Reproduced with permission.^[^ ^204^ ^]^ Copyright 2024, Wiley‐VCH. c) The electron transport properties of the SnO_2_ and SnO_2_‐FOA determined by the space‐charge‐limited‐current (SCLC). Reproduced with permission.^[^ ^210^ ^]^ Copyright 2023, Wiley‐VCH. d) Energy diagram of the formation of oxygen vacancies in TiO_2_ and TiO_2_/HFSTA. Reproduced with permission.^[^ ^213^ ^]^ Copyright 2024, Wiley‐VCH. e) Proposed schematic of enhanced hole extraction with PNRs. Reproduced with permission.^[^ ^223^ ^]^ Copyright 2021, American Chemical Society. f) UPS spectra of the bottom surface of perovskite films on PTAA and PTAA/API. Reproduced with permission.^[^ ^224^ ^]^ Copyright 2024, Wiley‐VCH.

We first discuss carrier transport regulation in PSCs with n–i–p structure. The extraction and transport of charge carriers at the buried interface are crucial for suppressing interface recombination and reducing carrier accumulation, which ultimately determine the efficiency and stability of PSCs. Similar to the strategy of strain control, the interlayer can also be used as an effective method to optimize the interfacial carrier transfer. In this vein, Xiong et al. reported that the chemical bonding between the perovskite and SnO_2_ layers can be achieved by introducing an interlayer of biguanide hydrochloride (BGCl).^[^ ^203^ ^]^ As shown in **Figure** **10**a, the Sn^4+^ ions in SnO_2_ interact with the nitrogen atoms in BGCl through coordination coupling, which establishes efficient pathways for electron transport between SnO_2_ and the perovskite layer. Moreover, the presence of Cl^−^ ions in BGCl facilitates the passivation of oxygen vacancies through electrostatic interactions, resulting in n‐type doping of the SnO_2_ layer. Consequently, this improved alignment of energy levels at the SnO_2_/perovskite interface enhances the abundance of electron donor species, leading to enhanced electron extraction and transport. Furthermore, the SnO_2_/perovskite interface can also be modified by inserting a 2D MBene interlayer, which induces a surface dipole moment and transfers extra electrons to the SnO_2_ surface.^[^ ^204^ ^]^ Consequently, the Fermi level of SnO_2_ is shifted upward, reducing energy losses at the interface and promoting efficient electron collection. In addition, 2D MBene significantly enhances the electrical conductivity of SnO_2_ from 3.89 × 10^−4^ to 5.61 × 10^−4^ S m^−1^ (Figure 10b), further enhancing electron transfer at the buried interface. Other materials, such as BTAC_4,_^[^ ^205^ ^]^ phosphorus‐containing Lewis acids,^[^ ^206^, ^207^ ^]^ fullerene derivatives,^[^ ^208^, ^209^ ^]^ and so on, have also been reported to facilitate charge transfer at the SnO_2_/perovskite interface.

Doping of the SnO_2_ layer is found to work in some cases, Dong et al. introduced FAI into SnO_2_ layers to control the carrier dynamics at the buried interface.^[^ ^194^ ^]^ The incorporation of FAI resulted in an upward shift of the conduction band and valence band of SnO_2_, creating a more favorable energy level alignment for efficient photocarrier transfer. FAI within SnO_2_ reacted with PbI_2_, facilitating perovskite crystallization. This process improved the morphology of the perovskite films and expanded the depletion region. The increased potential difference and widened depletion region at the buried interface enhanced carrier separation and sequential electron collection. Formamidine oxalate (FOA) has been discovered to exhibit a gradient distribution within the SnO_2_ layer, with a concentrated presence at the SnO_2_/perovskite interface.^[^ ^210^ ^]^ As illustrated in Figure 10c, FOA effectively enhances the electron mobility and surface potential of SnO_2_, enabling a better alignment of energy levels between SnO_2_ and the perovskite. This prevents carrier accumulation at the buried interface and improves the *V*_oc_ of PSCs. It is essential to point out that incorporating F^−^ into SnO_2_ significantly enhances its electron mobility and conductivity due to the strong interaction between F and Sn.^[^ ^174^, ^211^, ^212^ ^]^ As a result, the extraction and transfer of electrons at the SnO_2_/perovskite interface are accelerated, leading to a significant improvement in the *J*_sc_ of PSCs. Moreover, extensive research has focused on the surface modification of TiO_2_ ETL. For example, the heptadecafluorooctanesulfonate tetraethylammonium (HFSTA) interlayer reduces the occurrence of oxygen vacancies on the TiO_2_ surface (Figure 10d), which in turn lowers the surface energy of TiO_2_ and enhances the efficiency of carrier extraction.^[^ ^213^ ^]^ Interlayers such as CdS,^[^ ^214^ ^]^ 4‐chloro‐3‐sulfamoylbenzoic acid (CSBA),^[^ ^215^ ^]^ amorphous SnO_2,_^[^ ^216^ ^]^ and WO_3_^[^ ^127^, ^217^ ^]^ have also been used to adjust the carrier dynamics at the interface between TiO_2_ and perovskite.

In addition to n–i–p devices, much progress has also been made in carrier regulation of the buried interface in p–i–n PSCs. In early studies, PTAA was commonly used as the hole transport material, but it faced some challenges, such as bad wettability and low conductivity. It has been reported that the organic molecules such as 2PACz,^[^ ^218^ ^]^ 4‐butanediol ammonium Bromide (BD),^[^ ^219^ ^]^ sodium copper chlorophyllin (SCC),^[^ ^220^ ^]^ and polyvinyl oxide (PEO)^[^ ^221^ ^]^ are commonly used to modify the surface of PTAA in order to enhance its connection with perovskite and facilitate hole transport. It is worth noting that the 2, 4, 6‐tris(4‐aminophenyl)‐s‐triazine (TAPT) interlayer forms H‐π bonds between the amino group in TAPT and the benzene ring in PTAA.^[^ ^222^ ^]^ Additionally, the triazine group in TAPT forms π‐Pb^2+^ bonds with Pb^2+^ in the perovskite. These interactions effectively establish strong bonds between PTAA and the perovskite. Simultaneously, the presence of TAPT enhances the conductivity of PTAA, thereby facilitating the efficient hole transfer at the PTAA/perovskite interface.

Macdonald et al. introduced 1D phosphorene nanoribbons (PNRs) onto PTAA surface to enhance hole mobility.^[^ ^223^ ^]^ This leads to an accelerated extraction of holes at the PTAA/perovskite interface (Figure 10e), subsequently reducing hysteresis effects in the device and enhancing its photovoltaic performance. 1‐(3‐aminopropyl)‐imidazole (API) is introduced into PTAA/perovskite interface to enhance PSCs’ photovoltaic performance.^[^ ^224^ ^]^ The electron‐donating unit R‐C═N in API interacts with Pb^2+^, while R‐NH_2_ interacts with I^−^, both of which enhance the binding between PTAA and perovskite. Additionally, API also adjusts the energy level structure of the PTAA/perovskite interface (Figure 10f), thereby promoting interface electron transfer and improving the *J*_sc_ of the device. It is worth highlighting that Ph‐CH_2_N^+^H_3‐n_(CH_3_)_n_ constructed a charge carrier viaduct between PTAA and perovskite, which effectively manages the transport and extraction of carriers in both the in‐plane and out‐of‐plane directions.^[^ ^225^ ^]^

For the case of NiO_x_ modification, it is revealed that the presence of S in the (CH_3_)_3_SI interlayer triggers a conversion of the Ni element in NiO_x_, leading to an increased proportion of Ni^3+.[^ ^178^ ^]^ This increased Ni^3+^ content enhances the conductivity of NiO_x_, consequently accelerating the hole transport. Additionally, the formation of 1D (CH_3_)_3_SPbI_3_ optimizes the energy level alignment at the NiO_x_/perovskite interface, promoting efficient hole extraction and reducing the accumulation of carriers at the interface. Another interlayer 1,3‐bis(diphenylphosphino)propane (DPPP), was found to create hole transport channels between NiO_x_ and perovskite through the P‐terminated alkane chain.^[^ ^226^ ^]^ It has been widely reported that^[^4‐(3,6‐dimethyl‐9H‐carbazol‐9‐yl)butyl]phosphonic acid (Me‐4PACz) has been introduced onto the surface of NiO_x_ to facilitate the growth of large‐grain perovskite. These buried interface treatments contribute to improved charge extraction and reduced leakage current in PSCs. Another similar SAM material, (4‐(3,11‐dimethoxy‐7H‐dibenzo[c,g]carbazol‐7‐yl)butyl)phosphonic acid (MeO‐4PADBC), can establish stronger tridentate connections with NiO_x_, creating a more favorable surface for enhanced contact with the perovskite layer.^[^ ^227^ ^]^ Moreover, MeO‐4PADBC possesses an optimal dipole moment that facilitates the formation of an ideal energy level alignment between NiO_x_ and perovskite, thereby accelerating the hole transfer. Extensive research has also been conducted on buffer layers, such as 4‐hydroxyphenethyl ammonium halide,^[^ ^228^ ^]^ 2‐thiopheneethylammonium chloride (TEACl),^[^ ^229^ ^]^ 2‐mercapto‐1‐methylimidazole (MMI),^[^ ^230^ ^]^ and urea,^[^ ^231^ ^]^ to precisely modulate the energy level alignment at the interface between NiO_x_ and perovskite.

Various additives have been used to tune the hole transport at the NiO_x_/perovskite interface. For example, the cyanoacrylic‐acid‐based additive in the perovskite precursor can be spontaneously anchored onto the NiO_x_/perovskite interface during the crystallization of the perovskite.^[^ ^232^ ^]^ The carboxyl groups serve as a preferential pathway for hole transport, promoting the carrier's transfer at the buried interface. Another additive, 2‐aminoindan hydrochloride (AICl), can facilitate the formation of 2D/3D perovskite heterojunction from bottom to top, thereby controlling the interfacial carrier recombination and extraction dynamics at the NiO_x_/perovskite interface.^[^ ^16^ ^]^ Yu et al. reported that incorporating H_2_O_2_ into NiO_x_ increases the proportion of Ni^3+^ and forms NiOOH, which enhances the conductivity of NiO_x._^[^ ^233^ ^]^ This deliberate adjustment plays a crucial role in facilitating charge extraction at the buried interface and effectively suppressing non‐radiative recombination processes.

### 4.4 Inhibition of Adverse Reactions

The detrimental chemical reactions at the buried interface are the primary factors impacting the chemical stability of PSCs. Therefore, inhibiting such reactions is crucial to effectively enhancing device stability. Below, strategies are categorized based on the dominant reaction types at interfaces involving NiOx, TiO_2_, and PEDOT:PSS—three widely used CTLs.

**Figure 11:**
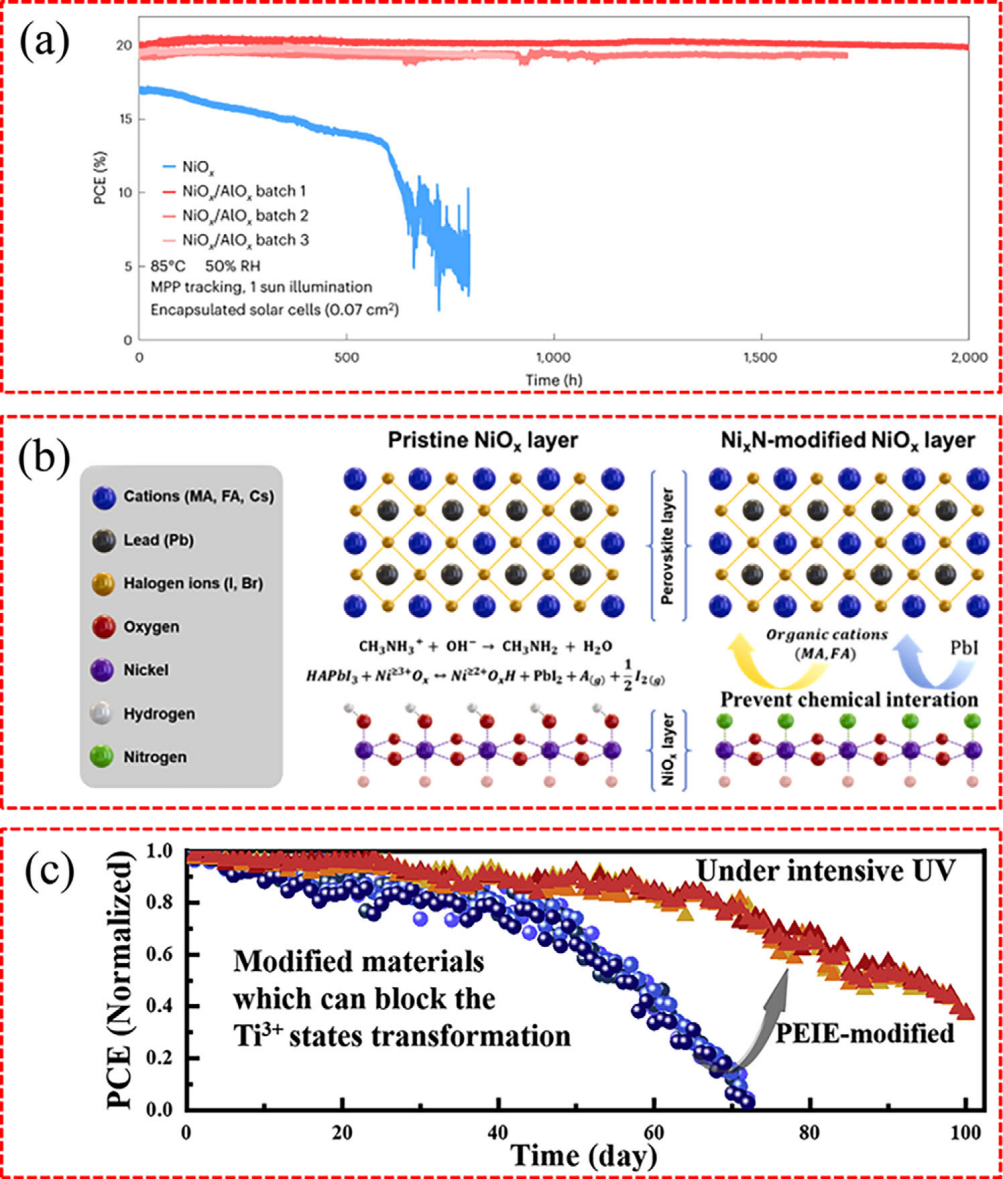
a) Long‐term thermal stability of the devices aged at 85 °C and 50% relative humidity under 1‐sun illumination. Reproduced with permission.^[^ ^235^ ^]^ Copyright 2024, Springer Nature. b) Schematic reaction of defect sites (Ni^≥3+^O_x_) and hydroxyl groups and perovskite that can occur on pristine and Ni_x_N‐modified NiO_x_ NPs thin films. Reproduced with permission.^[^ ^236^ ^]^ Copyright 2023, Elsevier. c) UV stability of the devices with and without PEIE modification. Reproduced with permission.^[^ ^131^ ^]^ Copyright 2020, Elsevier.

Me‐4PACz was used to prevent direct contact between NiO_x_ and perovskites.^[^ ^234^ ^]^ This significantly inhibits the adverse chemical reaction at the NiO_x_/perovskite interface, specifically Ni^3+^ + I^−^ → Ni^2+^ + I_2_, substantially enhancing the device's stability. Moreover, Me‐4PACz modified PSC maintains 97% of its initial efficiency even after aging for 1000 h at 80°C, and 84.6% of its original PCE after operating at the maximum power point (MPP) under continuous illumination for 1100 h. Yang et al. employed atomic layer deposition (ALD) to deposit a thin layer of AlO_x_ onto the surface of NiO_x._^[^ ^235^ ^]^ The acidic properties of AlO_x_ effectively prevent the deprotonation reaction between NiO_x_ and perovskite, resulting in a significant enhancement in the stability of PSCs. As shown in **Figure** **11**a, the AlO_x_‐modified PSCs exhibit no efficiency degradation even after continuous illumination operation at the MPP for 2000 h under the environment of 85°C and 50% relative humidity. Apart from AlO_x_ modification, NiO_x_ surface can also be modified by simply coating the nickel nitrate on NiO_x_ layer,^[^ ^236^ ^]^ which decreases the chemically active hydroxyl group and consequently enhancing the stability of PSCs (Figure 11b). The aprotic trimethylsulfonium bromide (TMSBr) buffer layer greatly enhances the stability of NiO_x_‐based PSCs.^[^ ^136^ ^]^ This enhancement can be attributed to several factors, including the excellent photothermal stability of TMSBr, the matched lattice constants between TMSBr and the perovskite layer. Most importantly, the positive charges of TMS^+^ positioned at the central S atom, and the neutral nature of the protons in the ─CH_3_ group. These characteristics effectively reduce the reactivity of deprotonation and degradation reactions.

The iodine‐terminated silane SAM was reported to serve as a barrier between the perovskite and TiO_2_ layer, effectively mitigating the photocatalytic degradation of perovskite induced by UV light.^[^ ^237^ ^]^ Hu et al. reported that F^−^ can reduce the photocatalytic activity of TiO_2_, thus significantly improving the UV stability of PSCs based on F‐TiO_2_. After continuous UV illumination for 26 h, F‐TiO_2_‐based PSCs still retain 68% of their initial efficiency.^[^ ^238^ ^]^ Guo et al. found that brookite TiO_2_ exhibits lower photocatalytic activity compared to anatase TiO_2_, thus leading to better UV stability in PSCs based on brookite TiO_2._^[^ ^239^ ^]^ It was reported that the adsorption of oxygen molecules on TiO_2_ can lead to the formation of superoxide radicals O_2_^−^, which is one of the main causes of photodegradation in PSCs.^[^ ^131^ ^]^ The introduction of a Ni_2_O_5_ buffer layer on the TiO_2_ surface can hinder the generation of O_2_^−^, greatly enhancing the photostability of TiO_2_‐based PSCs.^[^ ^240^ ^]^ It is worth mentioning that the polyethyleneimine ethoxylated (PEIE) modification layer can impede the transformation of Ti^3+^‐V_O_ states on the surface of TiO_2_, thereby disrupting the degradation pathway of PSCs. As shown in Figure 11c, the devices with PEIE modification, even after 72 days of exposure to UV light, still maintain 75% of their initial efficiency.^[^ ^131^ ^]^ Ito et al. discovered that the inclusion of Sb_2_S_3_ in the device can effectively suppress the I^−^/I_2_ reaction on the surface of TiO_2_, resulting in improved durability of the MAPbI_3_ layer against light exposure.^[^ ^130^ ^]^

Acidic PEDOT:PSS has been reported to react with the alkaline additive SnF_2_ in Sn‐based perovskite, thereby accelerating perovskite degradation. Zhou et al. introduced aqueous ammonia into PEDOT:PSS to control its acidity and suppress perovskite decomposition.^[^ ^241^ ^]^ The modified Sn‐based PSCs retained 91.3% of their original efficiency after 800 h of illumination. Arginine has also been reported to be used to adjust the pH value of PEDOT:PSS from 3.7 to 6.8.^[^ ^242^ ^]^ Adding NaOH can transform acidic PEDOT:PSS into an alkaline form, thereby enhancing PSCs’ stability. After being stored in a nitrogen environment for 1176 h, the NaOH‐modified Sn–Pb PSC exhibited remarkable retention of 93% of its initial efficiency.^[^ ^243^ ^]^ Coating the surface of PEDOT:PSS with iso‐pentylammonium tetrafluoroborate ([PNA]BF_4_) reduces its acidity and moisture absorption, effectively preventing the decomposition of perovskite films. The thermal stability of Sn–Pb PSCs was enhanced through^[^PNA]BF_4_ modification, as demonstrated by their successful storage at 85 °C for 240 h.^[^ ^244^ ^]^

In summary, inhibiting adverse chemical reactions relies on three core strategies: (1) physical isolation (e.g., SAMs, AlO_x_) to block reactant contact, (2) chemical passivation (e.g., F^−^ doping, acidity neutralization) to reduce active sites, and (3) defect engineering (e.g., PEIE modification) to disrupt degradation pathways. These approaches collectively enhance the chemical stability of buried interfaces, laying a foundation for durable PSCs.

### 4.5 Modifications of SAMs

**Figure 12:**
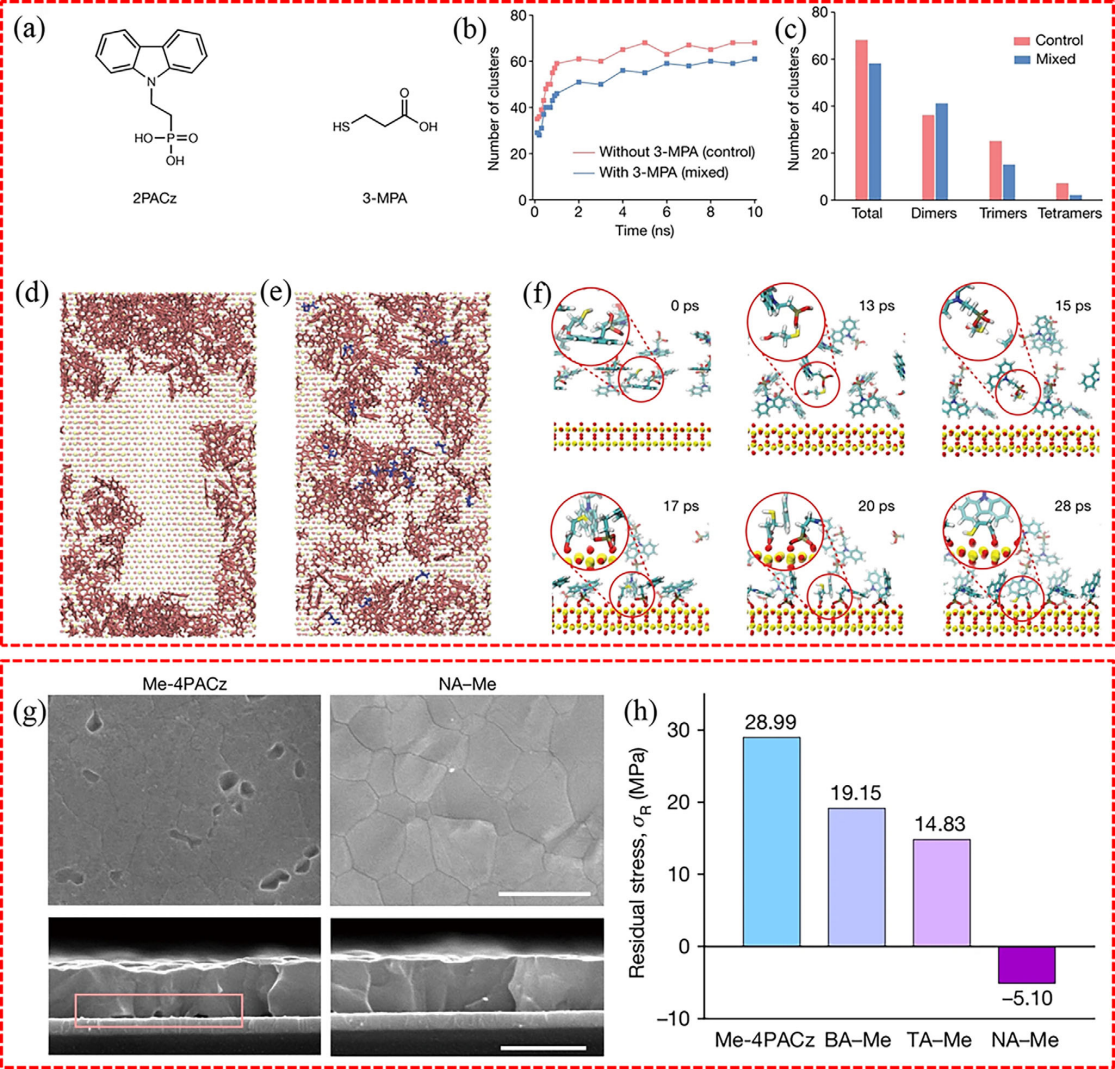
a) Chemical structures of the phosphonic acid 2PACz and the bifunctional compound 3‐MPA. b) Total number of 2PACz clusters formed over a set period, in the absence and presence of 3‐MPA. c) Types of 2PACz clusters formed at equilibrium. Top views of equilibrated molecular representations of control (d) and mixed (e) systems. 2PACz and 3‐MPA (where applicable) are shown in pink and blue, respectively; Sn and O atoms, shown in the background, are depicted in yellow and red, respectively. f) Successive steps along an AIMD trajectory showcasing the role of 3‐MPA as a co‐adsorbent. Large, encircled areas are magnifications of small ones. a–f) Reproduced with permission.^[^ ^67^ ^]^ Copyright 2023, Springer Nature. g) SEM images of the bottom surface of perovskite films on Me‐4PACz and NA‐Me SAMs. h) Comparison of residual stress in perovskite films on different HTLs. g,h) Reproduced with permission.^[^ ^247^ ^]^ Copyright 2024, Springer Nature.

In PSCs, the primary challenges with SAMs are their uneven coverage on the substrates (e.g., ITO, FTO), which introduces additional interfacial losses and device instability. On the one hand, phosphonic acid groups in SAMs tend to aggregate in solution, leading to incomplete substrate coverage.^[^ ^67^ ^]^ This non‐uniformity poses a critical hurdle for large‐area PSC manufacturing. On the other hand, weak interactions between SAMs and transparent conductive oxide (TCO) substrates cause partial detachment of SAMs during perovskite precursor coating,^[^ ^66^ ^]^ creating a significant stability issue that cannot be overlooked. To address these challenges, more precise control during SAM deposition is essential. For example, using molecular dopants or mixed‐solvent systems in SAM solutions can inhibit micelle formation. In simulations, Park et al. introduced 3‐mercaptopropionic acid (3‐MPA) (see **Figure** **12**a) to disrupt 2PACz clusters, particularly on textured substrates.^[^ ^67^ ^]^ This approach reduced cluster density on rough surfaces by 15% (Figure 12b) and decreased higher‐order clusters (trimers and tetramers) by 53% (Figure 12c), leading to less phase segregation and a dramatic increase in surface coverage (67% versus 15%, see Figure 12d‐f). The thiol group of 3‐MPA interacts with the phosphonic acid group of 2PACz, and the resulting supramolecular structure restricts the interaction between 2PACz molecules with their neighbors, thereby mitigating the formation of higher‐order clusters. When these two molecules reach the SnO_2_ surface, the interactions between 2PACz and SnO_2_, as well as between 3‐MPA and SnO_2_ become dominant over their intermolecular attractions. Liu et al. developed a co‐solvent strategy to disassemble carbazole‐based SAM micelles in processing solutions.^[^ ^245^ ^]^ This method increased the critical micelle concentration above the processing threshold, enhancing the reactivity of phosphonic acid anchoring groups and enabling dense SAM formation on ITO. Yu et al. used 5‐(9H‐carbazol‐9‐yl)isophthalic acid (CB‐PA) to modify the perovskite/MeO‐2PACz buried interface.^[^ ^246^ ^]^ CB‐PA chemically couples with MeO‐2PACz via π–π stacking and chelates with perovskite through double C═O···Pb bonds. Post‐assembly, CB‐PA fills voids in the MeO‐2PACz layer to form dense hybrid SAMs, creating a uniform surface potential and improved interfacial contact. As a result, defects at the buried interface are significantly passivated.

SAMs with surface‐rich nonpolar groups (e.g., long‐chain alkyl chains) exhibit low surface energy and weak interactions with polar solutions. This leads to solution agglomeration, uneven spreading, and formation of nonuniform perovskite films riddled with defects like pinholes and grain boundaries. To tackle this, common strategies include blending SAMs with other molecules or performing surface modification. Liu et al*. *reported a mixed SAM strategy where the widely used Me‐4PACz SAM is combined with the multi‐carboxylic acid‐functionalized aromatic molecule 4,4′,4″‐nitrotriphenyl acid (NA) to form a hybrid SAM (labeled NA‐Me) on NiO_x._^[^ ^247^ ^]^ Through π–π interactions between Me‐4PACz and the triphenylamine moiety of NA, the NA‐Me layer reduces Me‐4PACz aggregation, promoting uniform distribution of Me‐4PACz. This enhances carrier extraction and minimizes non‐radiative recombination at the NiO_x_/perovskite interface. As shown in Figure 12g,h, the presence of NA in the Me‐4PACz layer improves perovskite solution wettability on the hybrid surface layer, reducing nanoscale pinhole formation and relieving stress at the buried interface. Using this approach, inverted devices achieved an exciting PCE of 26.69%. In separate work, Pitaro et al*. *addressed infiltration issues with (2‐(3,6‐dibromocarbazol‐9‐yl)ethyl)phosphonic acid (Br‐2PACz) as a CTL by introducing a wetting layer of carbazole alkyl iodide derivative (4CzNH_3_I) atop Br‐2PACz, forming a self‐assembled bilayer.^[^ ^248^ ^]^ The driving force for bilayer formation is π–π stacking between the carbazole moieties of the two molecules. The ammonium iodide groups in 4CzNH_3_I coat the environmental interface, enhancing surface hydrophilicity and enabling deposition of a uniform perovskite layer. Notably, these ammonium iodide groups also act as a passivation layer at the buried interface, reducing surface defects that drive non‐radiative recombination. Zhang et al*. *developed an amphiphilic molecular CTL‐(2‐(4‐(dibenzylamino)phenyl)‐1‐cyanovinyl)phosphonic acid (MPA‐CPA)‐to create an ultra‐wettable bottom layer for perovskite deposition.^[^ ^22^ ^]^ By spin‐coating MPA‐CPA, they formed a bilayer structure on ITO comprising a chemically anchored surfactant layer and a disordered top layer. This top layer of randomly oriented MPA‐CPA molecules exhibited enhanced wetting properties for lead‐based perovskite precursor solutions.

**Figure 13:**
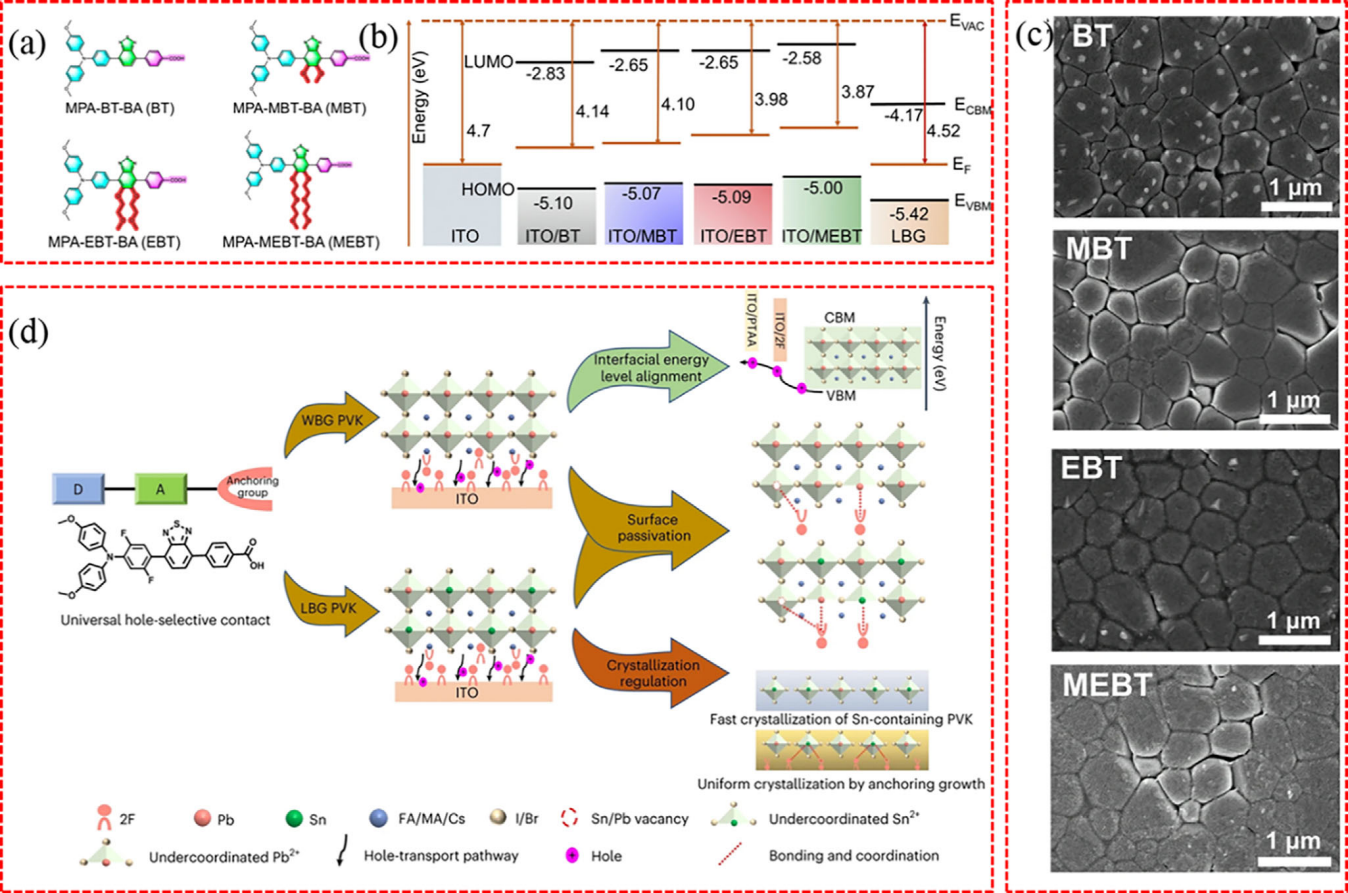
a) Molecular structure of BT, MBT, EBT, and MEBT. b) Energy level diagrams of the HSCs/perovskite. E_F_ and E_VAC_ correspond to Fermi and vacuum levels, respectively. E_CBM_ and E_VBM_ correspond to the energy of the valence band maximum and conduction band minimum, respectively. c) SEM images of buried interfaces of Sn–Pb perovskite films deposited on different HSCs. a–c) Reproduced with permission.^[^ ^249^ ^]^ Copyright 2025, Springer Nature. d) Interconnection mechanisms between ITO, 2F and perovskites. PVK, perovskite; VBM, valence band maximum; CBM, conduction band minimum; D, donor; A, acceptor. Reproduced with permission.^[^ ^250^ ^]^ Copyright 2023, Springer Nature.

Most SAMs are primarily applied to pure lead‐based devices. However, tin‐based PSCs‐widely used in all‐perovskite tandem cells‐pose new performance demands for SAMs due to their distinct bandgap widths. There is an urgent need to develop robust hole‐selective contacts (HSCs) for tin‐based PSCs and uncover their universal mechanistic principles. One strategy involves tuning SAM energy levels through structural modifications to match perovskite band alignments. In tin‐based PSCs, the acidic nature of polyaniline:polypyrrole and parasitic absorption losses often limit device performance and stability. Zhu et al. introduced oligoether side chains of varying lengths (methoxy, 2‐methoxyethoxy, 2‐(2‐methoxyethoxy)ethoxy) on benzothiadiazole units to synthesize target SAMs (**Figure** **13**a): MPA‐MBT‐BA (MBT), MPA‐EBT‐BA (EBT), and MPA‐MEBT‐BA (MEBT).^[^ ^249^ ^]^ As shown in Figure 13b, these oligoether side chains effectively regulate molecular HOMO and LUMO levels, enabling HSCs to accelerate hole extraction. In addition, they can also promote the uniform growth of perovskite grains (see Figure 13c), and passivate surface defects in tin‐lead perovskites. Notably, the optimized EBT‐LBG device achieved a peak efficiency of 23.54% due to improved tin–lead perovskite film quality and reduced interfacial non‐radiative recombination. This progress facilitated the construction of an all‐perovskite TSC with an efficiency of 28.61%. Another approach focuses on developing universal SAM materials. Zhu et al. designed the donor–acceptor molecule MPA2FPh‐BT‐BA (2F) to replace PTAA and PEDOT:PSS as an efficient HSC for diverse perovskite systems,^[^ ^250^ ^]^ including wide‐bandgap (WBG: 1.77 eV FA_0.8_Cs_0.2_Pb(I_0.6_Br_0.4_)_3_ and 1.68 eV FA_0.8_Cs_0.2_Pb(I_0.8_Br_0.2_)_3_), narrow‐bandgap (LBG: 1.25 eV FA_0.6_MA_0.3_Cs_0.1_Pb_0.5_Sn_0.5_I_3_), and standard‐bandgap (1.57 eV FA_0.8_Cs_0.2_Pb(I_0.95_B_0.05_)_3_) perovskites. As presented in Figure 13d, 2F exhibits compatibility with both wide‐bandgap (WBG) and low‐bandgap (LBG) perovskites, even when energy level alignment is suboptimal. In WBG devices, 2F accelerates hole extraction via energy level tuning and minimizes interfacial non‐radiative recombination by passivating defects. In LBG tin–lead devices, 2F slows crystallization kinetics and inhibits Sn^2+^ oxidation through stronger coordination and directional interactions with Sn^2+^ (versus Pb^2+^), improving film quality. This enabled all‐perovskite tandem devices to achieve a certified photovoltaic efficiency of 26.3% (with a measured peak of 27.22%).

### 4.6 Interface Modifications of the Textured Silicon for P/S‐TSCs

The industrially fabricated textured silicon bottom cells commonly feature pyramidal nanostructures at the micrometer scale. These pyramidal architectures manifest diminished reflectance losses attributable to the dual reflection phenomenon, thereby facilitating the absorption of incident light reflected by adjacent cones. However, for conventional 2T P/S‐TSCs, these nonuniform microstructures, exceeding the perovskite film thickness range, present substantial hurdles for solution‐processed fabrication of the defect‐free WBG perovskite films.^[^ ^251^ ^]^ The hybrid evaporation‐solution deposition protocol has been validated as a viable approach for fabricating high‐efficiency P/S‐TSCs.^[^ ^149^, ^252^ ^]^ This strategy entails sequential thermal evaporation of PbI_2_ and CsBr to engineer a homogeneous inorganic template, enabling precise nanoscale control over WBG perovskite film thickness. Subsequent solution‐phase treatment with organic cation salts further orchestrates the nucleation and crystal growth kinetics of WBG perovskites. While the hybrid two‐step evaporation‐solution methodology enables conformal perovskite film growth on textured silicon substrates, vacuum deposition of inorganic constituents restricts compositional tunability and impairs crystallization control compared to conventional all‐solution processing on polished or submicron‐textured substrates.^[^ ^150^, ^253^ ^]^

**Figure 14:**
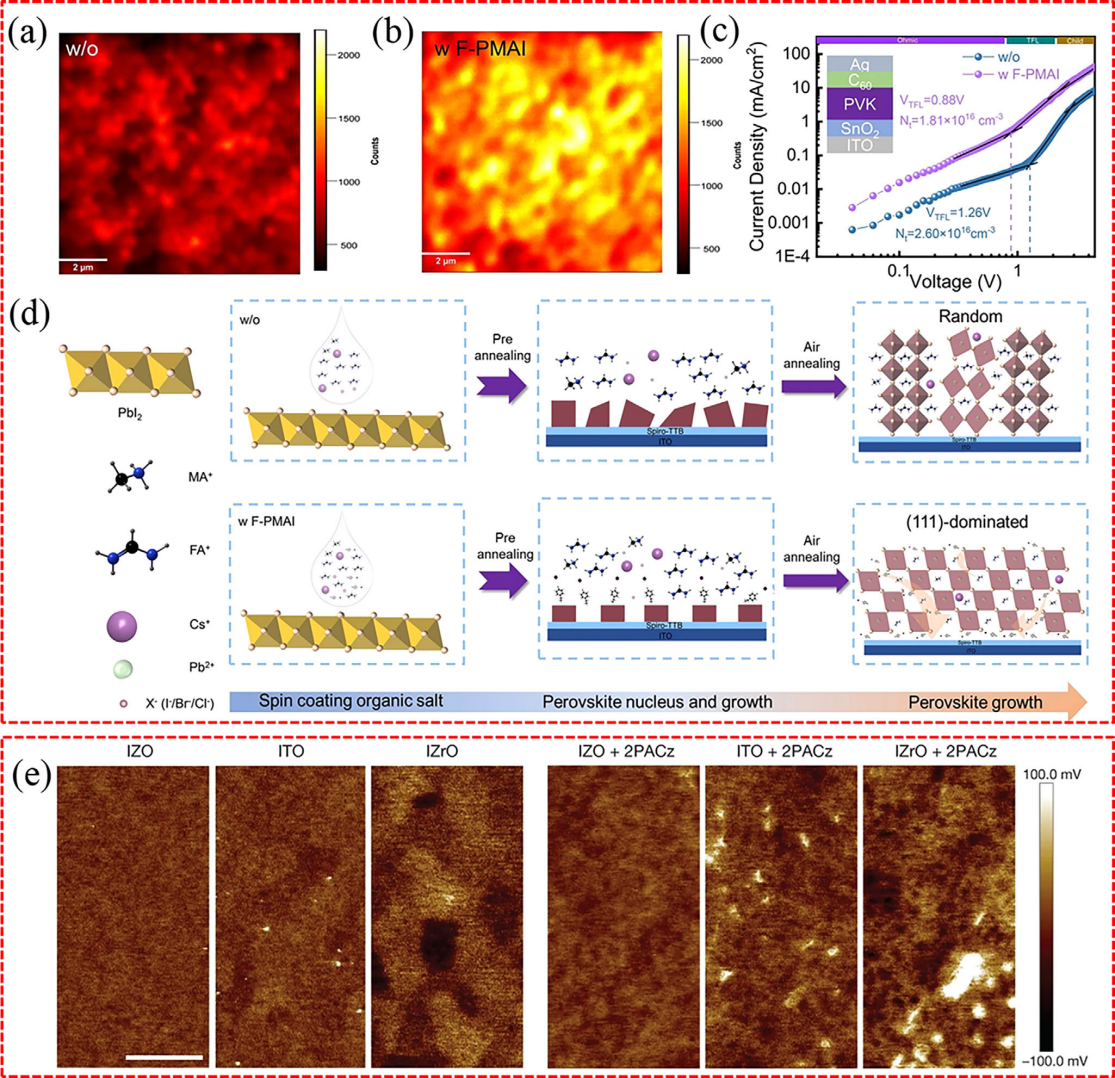
PL mapping of the perovskite films a) without and b) with F‐PMAI, with a structure of ITO/perovskite. c) SCLC curves of devices without and with F‐PMAI. d) Schematic illustration of the perovskite crystal growth process without and with F‐PMAI additive. a–d) Reproduced with permission.^[^ ^254^ ^]^ Copyright 2024, Springer Nature. e) KPFM surface‐potential mapping of 20‐nm‐thick interconnecting TCOs before (left) and after (right) 2PACz coverage. The scale bar (500 nm) applies to all KPFM images. Reproduced with permission.^[^ ^76^ ^]^ Copyright 2023, Springer Nature.

The evaporation‐assisted deposition method typically furnishes films characterized by suboptimal crystallinity and elevated defect density, particularly at the buried interface. In a bid to mitigate this challenge, a prevailing strategy entails the introduction of additives into the solution phase of evaporation‐assisted two‐step deposition to modulate the crystallization dynamics. For instance, Liu et al. introduced 4‐fluorobenzylammonium iodide (F‐PMAI) into an organic salt solution and fabricated high‐quality perovskite films with superior structural regularity via a hybrid evaporation‐solution protocol.^[^ ^254^ ^]^ This outcome was attributed to the F^−^ moieties establishing hydrogen‐bonding interactions with organic cations, which retarded perovskite crystal growth kinetics‐thereby promoting grain coarsening and defect reduction (see **Figure** **14**a–c). As shown in Figure 14d, F‐PMAI molecules modulate the surface energy of (111) facets, thereby preferentially promoting (111)‐plane epitaxy. Post‐growth, these amphiphilic molecules accumulate on both the buried and top surfaces of the perovskite film‐synergistically enhancing charge carrier transport and interfacial passivation. Furthermore, a panoply of additives, including MA(Cl_0.5_SCN_0.5_),^[^ ^150^ ^]^ thioacetylacetamide chloride (TAACl),^[^ ^255^ ^]^ and CdAc_2_^[^ ^256^ ^]^ have demonstrated effect in ameliorating film quality during evaporation‐assisted deposition on textured silicon substrates, thereby underscoring the generality of this approach.

**Figure 15:**
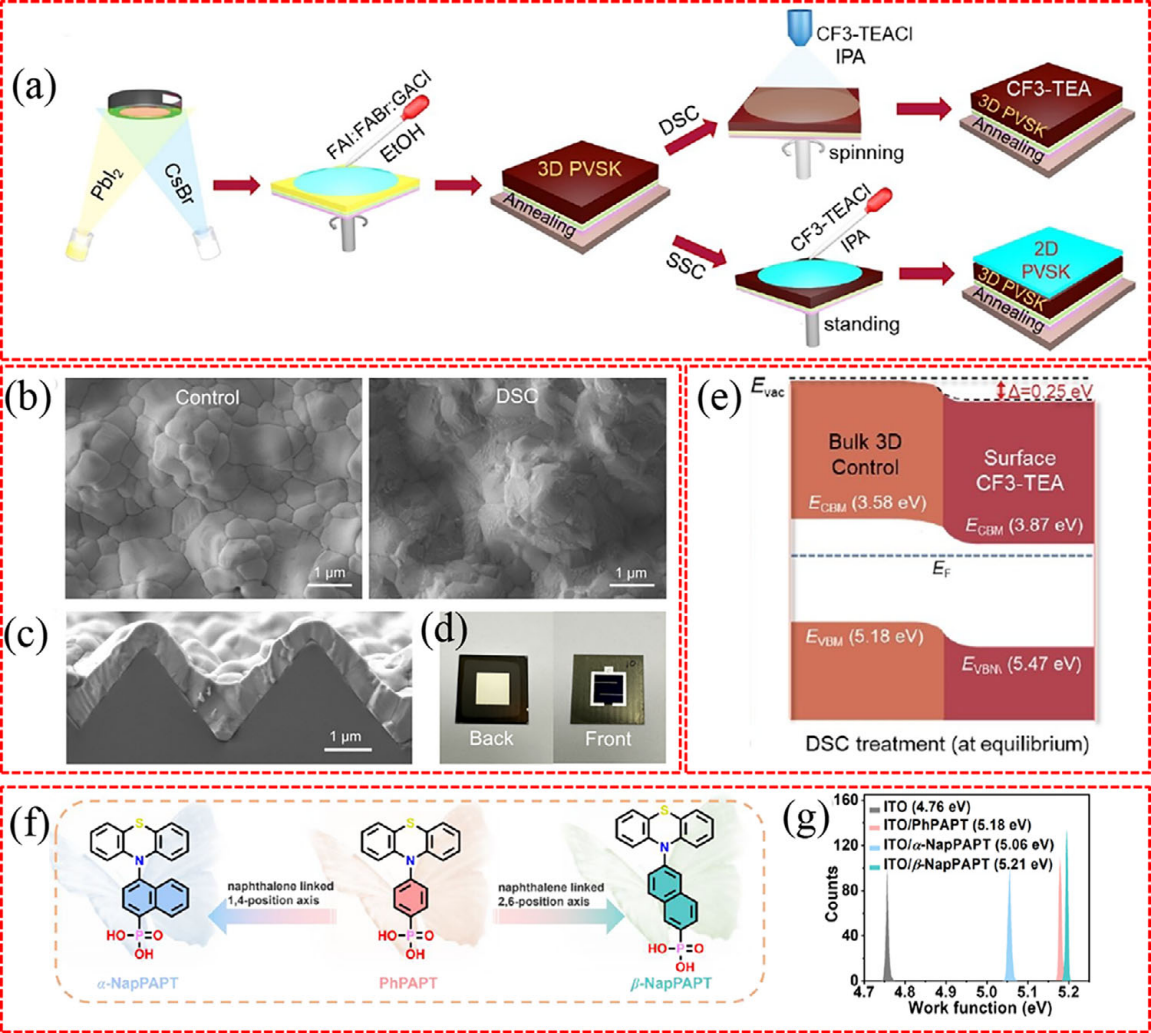
a) Schematic diagram of the process of the perovskite film preparation, including the hybrid two‐step fabrication of the perovskite films and the DSC (SSC) surface treatment. b) SEM topview images of the control and DSC perovskite films. c) Cross‐section images of the perovskite film on a textured c‐Si. d) The photographs of a tandem cell fabricated on a textured silicon solar cell. e) Surface energy levels of the DSC‐treated perovskite films and the corresponding spatial band‐bending schematics at equilibrium. a–e) Reproduced with permission.^[^ ^258^ ^]^ Copyright 2024, Wiley‐VCH. f) Molecular structures and design strategy of PhPAPT, *α*‐NapPAPT, and *β*‐NapPAPT. g) The work function distribution of bare ITO, PhPAPT, *α*‐NapPAPT, and *β*‐NapPAPT‐treated ITO surfaces. f,g) Reproduced with permission.^[^ ^260^ ^]^ Copyright 2025, Wiley‐VCH.

In single‐junction PSCs, buried interface interlayers have emerged as a vanguard strategy to regulate crystal growth kinetics, fine‐tune perovskite grain morphology, and eliminate interfacial defects in overlying perovskite layers. Notably, this strategy also demonstrates efficacy on textured silicon substrates. Given the inherent heterogeneity of solution‐based methods on textured substrates, vapor deposition is frequently employed to engineer buried interlayers. For instance, Zhang group proposed leveraging 2D perovskite with cross‐linkable ligands to fabricate a robust buried interface interlayer.^[^ ^257^ ^]^ They first thermally evaporated an ultrathin PbI_2_ film, which was subsequently converted to a 2D perovskite framework via sequential spin‐coating of 4‐vinylbenzylammonium iodide (VBAI). Photon‐induced cross‐linking of vinyl moieties then engendered covalent bond formation within the (VBA)_2_PbI_4_ lattice, yielding a mechanically robust 2D perovskite matrix. This structurally ordered interface layer provides advantages through preferential crystal growth of strain‐free, uniform upper perovskite layers. It mitigates interface defect‐induced instability and recombination, while facilitating charge carrier extraction via optimal energy level alignment. The bottom‐contact heterostructure has demonstrated wide‐ranging applicability‐enabling conformal coverage of diverse textured substrates and compatibility with various precursor solutions that remain intact without erosion. Aydin et al. addressed the long‐standing challenge of achieving uniformly coated SAMs on oxide substrates by utilizing an ultrathin (5 nm) grain‐free indium zinc oxide (IZO) as a TCO interlayer.^[^ ^76^ ^]^ Compared to conventional polycrystalline TCOs, IZO exhibits exceptional homogeneous surface potential due to its lack of grain boundaries (see Figure 14e), along with a higher density of surface active sites. By integrating the optical enhancement from an isometrically thin IZO back electrode and a meticulously optimized front‐layer contact structure, the study achieved an independently verified PCE of 32.5% for P/S‐TSCs. As shown in **Figure** **15**a, the dynamic spray coating (DSC) method has proven to be a viable approach. Figure 15b, c demonstrates that fluorinated thienylethylammonium (CF_3_‐TEA) enables uniform surface coverage and effectively suppresses phase transitions on textured substrates.^[^ ^258^ ^]^ The incorporation of trifluoromethyl moieties enhances surface passivation by facilitating dipole layer formation‐achieved through strong intermolecular interactions and precise energy level alignment (see Figure 15e).

Unlike perovskite films that exhibit thickness inhomogeneity on textured substrates, SAMs form uniform covalently bonded layers on oxide substrates. This suppresses material accumulation at the base of pyramidal topographies, enabling solution‐processed deposition. Nevertheless, fabricating SAMs with precise monolayer thickness and high packing density remains a significant challenge, driving concerted efforts toward hierarchical optimization of SAM architectures. Er‐Raji et al. precisely regulated the annealing temperature of SAMs and found that when the annealing temperature of SAMs increased from 100 °C to 150 °C, the thickness of SAMs could decrease from 5 nm to a monolayer.^[^ ^259^ ^]^ In addition, high‐temperature conditions promote dense adsorption of SAMs on the metal oxide surface, thereby enhancing the passivation quality of the SAM/perovskite interface in P/S‐TSCs. The packing arrangement of SAMs is highly correlated with the inherent properties of SAM materials. As shown in Figure 15f, Li group designed a series of benzothiazole‐based aromatic SAMs to elucidate how diverse aromatic linkers modulate molecular packing, substrate work function tuning, and carrier transport kinetics.^[^ ^260^ ^]^ The study revealed that naphthyl aromatic linkers along the 2, 6‐position axis (β‐Nap) enable the construction of dense and highly ordered carrier‐selective interfaces, enhancing interfacial interactions and facilitating ideal energy level alignment with perovskite films (see Figure 15g). This strategy enabled perovskite/silicon tandem structures to achieve an efficiency of 28.89%.

## 5 Summary and Outlook

Buried interfaces in PSCs are pivotal determinants of device performance and stability, governing processes from perovskite crystallization and carrier transport to chemical degradation. This review has systematically elaborated on the diverse challenges at these interfaces, including defects, residual strain, inefficient carrier dynamics, adverse chemical reactions, and specific issues in SAM‐based devices and P/S‐TSCs. Corresponding modification strategies—such as defect passivation, strain control, carrier transport regulation, and inhibition of harmful reactions—have been summarized, highlighting their roles in mitigating interfacial losses and enhancing device efficiency and operational stability.

Despite significant progress, several critical limitations persist, demanding targeted efforts to advance buried interface engineering:

1) Deficiency in Dynamic, In Situ Characterization:Current understanding of buried interfaces relies heavily on ex situ techniques (e.g., XPS, SEM), which fail to capture real‐time evolution under operational stress (e.g., light, heat, humidity). Key technical bottlenecks include the inability to achieve both high spatial resolution (nm scale) and temporal resolution (µs scale) in complex operational environments, as well as limited penetration depth for probing buried interfaces beneath thick functional layers. For instance, defect migration, strain relaxation, and chemical reactions at the interface are highly dynamic processes, yet their transient behaviors remain poorly resolved. Operando XRD can real‐time monitor strain evolution in perovskite films under illumination or thermal stress by tracking shifts of Bragg peaks, directly correlating lattice deformation (e.g., tensile strain‐induced bandgap widening discussed in Section 3.3) with device performance degradation. Additionally, TRPL enables in situ tracking of carrier dynamics at the interface, capturing changes in recombination lifetime to reflect charge extraction efficiency variations under operational conditions. Future research must prioritize developing advanced in situ characterization tools, such as operando X‐ray diffraction (XRD) for strain tracking, time‐resolved photoluminescence (TRPL) for carrier dynamics, and in situ X‐ray photoelectron spectroscopy (XPS) for chemical state monitoring, to unravel the interplay between interfacial changes and device degradation.
2) Limitations in Material Design and Compatibility:While SAMs and novel CTLs have improved interface quality, their long‐term compatibility with perovskites remains underexplored. For example, SAMs often suffer from molecular aggregation or desorption under prolonged illumination, and metal oxide CTLs (e.g., NiO_x_, TiO_2_) still exhibit inherent defects (e.g., oxygen vacancies) that trigger adverse reactions. Future work should focus on designing multifunctional materials: i) CTLs with tunable lattice parameters and thermal expansion coefficients to minimize strain; ii) hybrid interlayers (e.g., metal–organic frameworks, 2D materials) that simultaneously passivate defects, regulate carrier transport, and block chemical reactions; iii) given the recent prevalence and effectiveness of SAMs, emerging directions include multifunctional molecular design integrating defect passivation, energy level tuning, and water/oxygen barrier properties, synergistic integration with 2D materials or metal–organic frameworks to enhance charge transport and mechanical robustness and scalable synthesis of SAMs with uniform monolayer coverage for large‐area modules, addressing aggregation issues in roll‐to‐roll processing.
3) Challenges in Scaling and Tandem Device Integration:Practical application of PSCs, particularly in P/S‐TSCs, is hindered by the inability to achieve uniform perovskite deposition on textured silicon substrates. Critical challenges include: i) non‐uniformity of interface modification over large areas (e.g., >100 cm^2^), leading to localized strain accumulation and defect aggregation at edges or grain boundaries; ii) compatibility issues between large‐area deposition techniques (e.g., roll‐to‐roll coating) and interface modifiers, which may degrade or desorb during high‐speed processing; iii) amplified interfacial degradation in modules due to prolonged exposure to environmental stress (moisture, heat) over extended interfaces, exacerbating ion migration and chemical reactions at buried interfaces. Current strategies (e.g., evaporation‐solution hybrid methods) improve conformal growth but complicate manufacturing processes. Scalable techniques, such as dynamic spray coating or inkjet printing, require optimization to control perovskite nucleation on microscale pyramidal textures. Additionally, the mismatch in energy levels and charge extraction kinetics between perovskite and silicon layers remains a bottleneck; future studies should focus on interface engineering to balance photocarrier collection and minimize recombination at the tandem junction.
4) Overlooking Long‐Term Stability Mechanisms:Most studies evaluate stability over short periods (≤1000 h), with limited insight into degradation mechanisms at the buried interface over device lifetimes. For example, ion migration (e.g., I^−^ diffusion into CTLs) or interfacial phase segregation may accelerate over time, even in initially stable devices. Future research must emphasize long‐term aging tests combined with post‐mortem analysis to identify failure modes, such as: i) chemical degradation products at the interface (e.g., PbI_2_, metal halides); ii) morphological changes (e.g., void formation, grain boundary coarsening); iii) shifts in energy level alignment. These insights will guide the design of “self‐healing” interfaces (e.g., modifiers that regenerate passivation sites or block ion diffusion pathways).
5) Need for Interdisciplinary Collaboration:Buried interface engineering requires integrating materials chemistry, device physics, and computational modeling. For instance, DFT simulations have predicted favorable terminations and defect passivation mechanisms, but experimental validation lags due to the complexity of replicating theoretical models. Strengthening collaboration between theorists and experimentalists will accelerate the rational design of interfaces—e.g., using machine learning to screen potential modifiers based on binding energy and defect passivation efficiency, then validating their performance in devices. Moreover, phase‐field simulations can model perovskite crystallization kinetics at buried interfaces, predicting how interfacial energy and lattice mismatch affect grain growth and defect distribution, thereby guiding the optimization of deposition processes for uniform, strain‐free films. These interdisciplinary tools bridge computational prediction and experimental validation, enabling more efficient exploration of interface engineering strategies.

In conclusion, optimizing buried interfaces is indispensable for bridging the gap between lab‐scale PSC performance and commercialization. By addressing the limitations in characterization, material design, scalability, and stability, and leveraging interdisciplinary approaches, future research can unlock the full potential of PSCs as a high‐efficiency, low‐cost photovoltaic technology.

## Conflict of Interest

The authors declare no conflict of interest.
